# Stabilizing sub-nanoporous zinc metal–organic frameworks in SnTe thermoelectrics for high-temperature power generation

**DOI:** 10.1039/d6sc04137j

**Published:** 2026-06-22

**Authors:** Wang Yue, Decheng An, Jiali Ren, Fangyuan Chen, Zhijun Hao, Jiaxi Song, Zejin Li, Wutao Yang, Shaoping Chen, Yuan Yu, Xian-Ming Zhang

**Affiliations:** a College of Chemistry and Chemical Engineering, State Key Laboratory of Clean and Efficient Coal Utilization, Analysis Testing and Equipment Sharing Center, Taiyuan University of Technology Taiyuan 030024 China andecheng@tyut.edu.cn zhangxianming@tyut.edu.cn; b Institute of Physics (IA), RWTH Aachen University Sommerfeldstraße 14 Aachen 52074 Germany yu@physik.rwth-aachen.de

## Abstract

The integration of porous metal–organic frameworks (MOFs) with thermoelectric materials offers an effective route toward the phonon-glass electron crystal paradigm. However, their application in high-temperature power generation remains severely limited by the intrinsic thermal instability of MOFs arising from weak coordination bonding. Here, we report a thermally robust heterostructure that is stable from 300 to 873 K, achieved by embedding sub-nanoporous Zn-[2-methylimidazolate] frameworks (ZIF-8) into Sn-rich SnTe nanomaterials synthesized by a base-amine-mediated solvothermal method. We reveal that the remarkably low solubility of Zn in SnTe inhibits Zn diffusion, thereby stabilizing the Zn nodes and preserving the Zn–N coordination network at elevated temperatures. The SnTe/ZIF-8 nanocomposite introduces an interface-induced energy filtering effect for electrical optimization. Simultaneously, the porosity-induced thermal radiation, the hierarchical defects, and the interfacial Kapitza resistance enable strong phonon scattering across a broad frequency spectrum. As a result, a maximum *zT* value of 1.3 at 873 K is achieved, among the highest values reported for heterostructure-enhanced SnTe thermoelectric materials without lattice doping. This work provides essential design principles for thermally stable MOF-based heterostructures and extends their applicability to high-temperature (>773 K) thermoelectric power generation.

## Abstract 2

The integration of porous metal–organic frameworks (MOFs) with thermoelectric materials offers an effective route toward the phonon-glass electron crystal paradigm.

## Graphical abstract

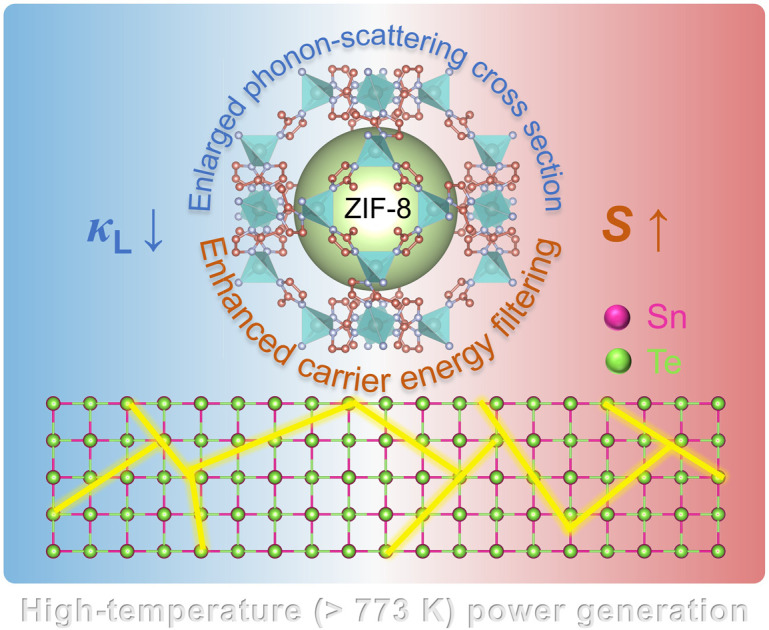

## 1 Introduction

Thermoelectrics (TEs) can directly convert waste heat into electricity without hazardous emissions, offering a promising solution for relieving the energy crisis and environmental pollution.^1–3^ The thermoelectric conversion efficiency is determined by a dimensionless figure of merit *zT* = (*S*^2^/*ρT*)/*κ*_tot_, where *S*, *ρ*, *κ*_tot_, and *T* represent the Seebeck coefficient, electrical resistivity, total thermal conductivity, and absolute temperature, respectively. To achieve higher *zT* values, it is essential to strike a careful balance between improving the power factor (PF = *S*^2^*/ρ*) and minimizing *κ*_tot_ (*κ*_tot_ = *κ*_e_ + *κ*_L_; contributed by electrons, *κ*_e_, and phonons, *κ*_L_). This trade-off regarding phonon-glass electron-crystals^4^ is fundamentally governed by the interconnected nature of charge carrier and heat transport mechanisms, which has spurred considerable efforts for developing efficient strategies for decoupling electronic and phononic transport pathways, including carrier concentration tuning,^2^ band structure engineering,^5,6^ and nanostructuring.^7,8^ To date, these means are routinely based on the modification of the matrix lattice by extrinsic doping and/or thermodynamic regulation of crystal growth.^7,9^ Recently, structural defects, such as grain boundaries,^10–12^ precipitates,^2,13^ and nanocomposite coatings,^14,15^ have been increasingly found to play an indispensable role in controlling the transport of heat and charge.

Introducing pores into the microstructure is another useful strategy to strongly scatter phonons.^16,17^ Additionally, porosity might not inevitably block carrier transport if these pores are on the nanoscale and the matrix forms a conductive cross-linked network.^18,19^ Metal–organic frameworks (MOFs) are representative porous materials, which are composed of a network of metal ions linked to organic ligands by coordination bonds.^20^ They generally possess ultrahigh porosity and surface areas as well as long-range translational symmetry.^19,21,22^ The pores are periodically distributed and exhibit diameters typically ranging from 0.5 (ultramicropores) to 3 nm (mesopores). However, little is known about the enhanced thermoelectric properties of MOFs mainly because it is a great challenge to ensure the favorable stability of MOFs in the high-temperature regime, in particular, the breakthrough of the *ca.* 773 K barrier.^23^ Obtaining stable thermoelectrics at high temperatures is essential for durable applications.^24^ To the best of our knowledge, thermal decomposition of MOFs is, in most cases, a result of node–linker bond breakage.^25^ The initial instability is either inorganic-node-centred or ligand-centred.^23^ Even the combination of low node stability and high ligand stability also leads to unstable coordination environments. Among MOFs, ZIF-8 (zinc(ii)2-methylimidazolate) stands out with good thermal stability due to its robust Zn–N coordination bonds (with ionic–covalent bond nature^26^) established between the Zn^2+^ ions and the anionic 2-methylimidazole ligands.^27,28^ Also, the 2-methylimidazole entity possesses good thermal stability because of the presence of strong H-bonding interactions between methyl ‘H’ atoms and ring ‘N’ atoms present in it. Theoretically, as long as the Zn node does not dissolve in the thermoelectric matrix, the resulting stable Zn–N coordination can ensure that ZIF-8 avoids the remarkable collapse of its framework upon thermomechanical processing. These should become the design criteria for thermally stable thermoelectric/MOF heterostructures.

Fortunately, a large number of studies have found that Zn has extremely low solubility in SnTe regardless of high temperature.^29–32^ Moreover, SnTe, as a top Pb-free alternative to PbTe, possesses many thermoelectric-favorable properties such as large valley degeneracy and strong lattice anharmonicity, attributed to its metavalent bonding nature.^33,34^ The valence electrons in metavalent bonding are in a competitive state between localization and delocalization,^35^ which enables the SnTe system to be characteristic of incipient metals with high electrical conductivity. Yet, the room-temperature *κ*_L_ of SnTe (∼3 W m^−1^ K^−1^) is still much higher than its amorphous minimum,^30^ requiring the introduction of defects for further scattering of heat-carrying phonons. Some density functional theory (DFT) studies have demonstrated that more than 50% of the *κ*_L_ of SnTe is contributed by phonons with a mean free path (MFP) below 10 nm, implying that the construction of sub-nano and/or nano defect structures can ensure sufficient *κ*_L_ reduction.^36,37^ These findings raise a critical question for MOF chemistry in incipient metal SnTe: can a porous ZIF-8 heterogeneous layer be stably constructed at SnTe grain boundaries to further reinforce its phonon-glass electron-crystal characteristics?

Here, we present a pioneering study on MOF-enhanced high-temperature (up to 873 K) thermoelectrics. Along the total chemical synthesis route, we first develop a cost-effective solvothermal synthesis method for the production of SnTe nanocrystals (NCs). By utilizing a dimethylformamide–sodium hydroxide–sodium borohydride solution system, elemental Sn is successfully reduced and well-maintained in NCs, enabling the optimization of matrix carrier concentration (∼1.25 × 10^20^) *via* the Sn self-compensation mechanism. By combining multiscale spectroscopic, microscopic, thermodynamic, and mechanical characterization studies, we discover that sub-nano porous ZIF-8 can be stabilized from 300 to 873 K *via* creation of a heterostructure in SnTe thermoelectric nanomaterials. This unique architecture of SnTe/*x*wt%ZIF-8 (*x* = 0, 1, 2, 3, 4, and 5) nanocomposites containing a cross-linked matrix network and multi-level interfacial defects allows for thermal radiation, multiscale phonon scatterings and interfacial Kapitza resistance along with well-maintained carrier weighted mobility, and engenders an extremely low *κ*_L_ of ∼0.18 W m^−1^ K^−1^ at 873 K that is even lower than the value predicted by a diffusion-mediated transport model.^38^ In addition, dual incorporation of trace organic species on the SnTe NC surface and large bandgap ZIF-8 induces a dual-barrier-driven energy filtering effect, and thus maximizes *S* and the power factor. Profiting from these two positive aspects, a peak *zT* value of ∼1.3 at 873 K and a single-leg power density *P*_out_ of ∼130 mW cm^−2^ at Δ*T* = 463 K are achieved for SnTe/3wt%ZIF-8, comparable to the state-of-the-art *zT*values for heavily doped SnTe.^6–8,39–42^ This finding advances the mechanistic understanding of heterostructure engineering in thermoelectrics, highlighting interfacial chemistry not merely as a secondary tuning parameter, but as a fundamental design lever for simultaneously modulating electronic and phononic transport.

## 2 Results and discussion

### 2.1 Large-scale construction of self-compensated SnTe and porous ZIF-8 NCs

**Fig. 1:**
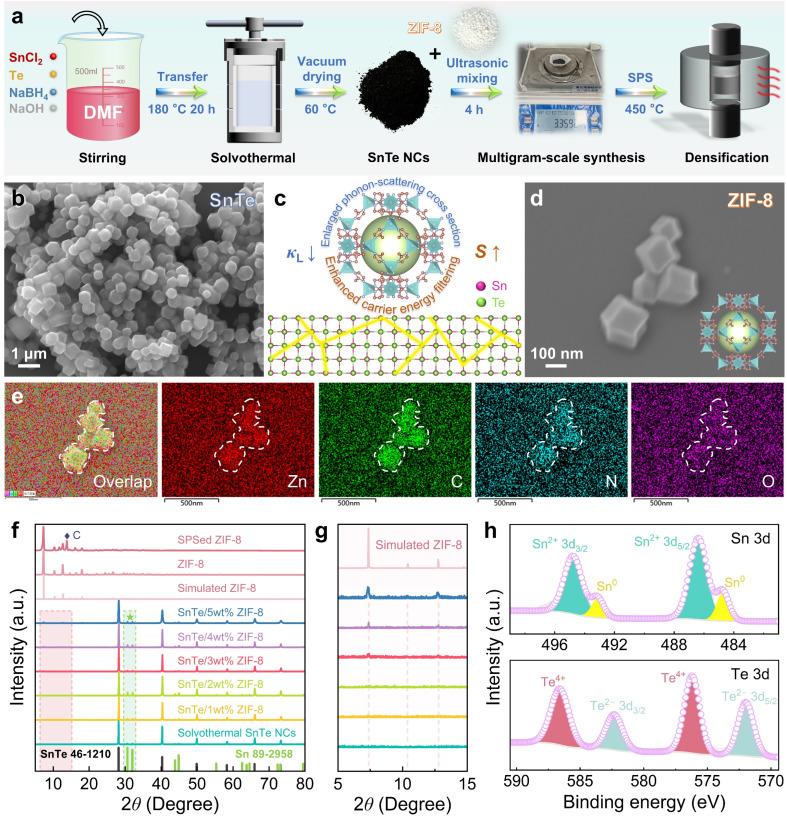
(a) Schematic overview of the synthesis process of SnTe/ZIF-8 nanocomposites. (b) SEM image showing the morphology of solvothermal-synthesized Sn-rich SnTe nanocrystals. (c) Design schematic diagram illustrating the carrier–phonon decoupling effect in SnTe/ZIF-8 hetero-composites. (d) SEM and (e) EDS results of ZIF-8 nanocrystals, (d) SEM and (e) EDS results of ZIF-8 nanocrystals, showcasing 12 {110} facets. A unit cell of ZIF-8 is shown in the inset of (d). (f) XRD patterns of ZIF-8, as-SPSed ZIF-8, and as-SPSed SnTe/*x*wt%ZIF-8 (*x* = 0, 1, 2, 3, 4, and 5). Sharp and strong XRD peaks indicate good crystallinity of the products. Bits of carbon species in the as-SPSed ZIF-8 pattern may come from the imidazole/azirine-containing residue resulting from fragmentation of the methylimidazole linkers.^44^ (g) Expanded XRD patterns showing the visible ZIF-8 phase with increasing *x*. (h) High-resolution Sn 3d and Te 3d XPS spectra of solvothermal SnTe nanocrystals. The main peaks at higher binding energies in the Te 3d region correspond to Te^4+^. According to the known tellurium chemistry,^40^ the observed Te(ii) and Te(iv) surface oxides obey the following evolution mechanisms: 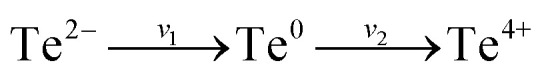. There is measurable Te^4+^ accumulation at the surface when the rate *v*_2_ < *v*_1_. These findings are different from those observed in the familiar liquid-phase synthesis of metal telluride crystals containing Te^0^ byproducts,^41–43^ where the telluride ion (Te^2−^) cannot remain stable because of the absence of tin ions and is oxidized to tellurium (Te^0^).

To fulfill the demands for practical thermoelectric applications, we enable the large-scale synthesis of SnTe/*x*wt%ZIF-8 (*x* = 0, 1, 2, 3, 4, and 5) nanocomposites using a two-step method, as schematically illustrated in Fig. 1a. Specifically, we first develop a cost-effective, repeatable, and surfactant-free solvothermal approach of preparing SnTe nanocrystals (NCs) on a gram-scale (the yields of the products are >3 g per autoclave). The scanning electron microscopy (SEM) micrograph of the as-prepared SnTe showed a regular cubic morphology with an edge length of *ca.* 450 nm (Fig. 1b and S1a). Powder X-ray diffraction (PXRD) patterns (Fig. 1f and S2, Discussion 1 in the SI) of the produced SnTe NCs revealed a matrix phase of the rock-salt structure (space group *Fm*3̄*m*; PDF# 46-1210). Besides, conspicuous secondary-phase Bragg reflections indexed to tetragonal metallic Sn (PDF# 89-2958) can be observed. Employing X-ray photoelectron spectroscopy (XPS) measurements, we further characterized the chemical state of these SnTe NCs, as shown in Fig. 1h and S3. The Sn 3d spectrum clearly shows two prominent peaks at 494.8 eV and 486.4 eV associated with a Sn^2+^ oxidation state^6^ (corresponding to Sn 3d_3/2_ and Sn 3d_5/2_, respectively) within a chalcogenide chemical environment. The weak shoulders at 493.1 eV and 484.7 eV are attributed to elemental Sn^0^, indicating a Sn self-compensated SnTe lattice. The Te 3d spectrum reveals the presence of two different oxidation states (Te^2−^ and Te^4+^) for tellurium atoms. The spin doublets (3d_5/2_ and 3d_3/2_), present at 571.9 and 582.3 eV, respectively, are in good agreement with the reported values for Te^2−^.^39^ The elemental Sn^0^ can suppress the Sn vacancies in SnTe NCs and, in turn, manipulate their thermoelectric properties (discussed later). The porous ZIF-8 NCs were prepared according to the established synthetic protocol.^43^ As shown in Fig. 1d and S1b, the synthesized ZIF-8 possesses a truncated rhombic dodecahedral shape with a ∼380 nm size. The energy-dispersive X-ray spectroscopy (EDS) elemental mapping images indicate the uniform distribution of Zn, C, N, and O in the ZIF-8 particles (Fig. 1e). The XRD pattern of the obtained ZIF-8 matches well with the simulated peaks located at 7.28°, 10.32°, and 12.67° (2*θ*), corresponding to the (110), (200), and (211) diffractions, respectively (Fig. 1f). These results confirm the successful synthesis of ZIF-8.

### 2.2 Existence of thermally stable SnTe/ZIF-8 heterostructures

**Fig. 2:**
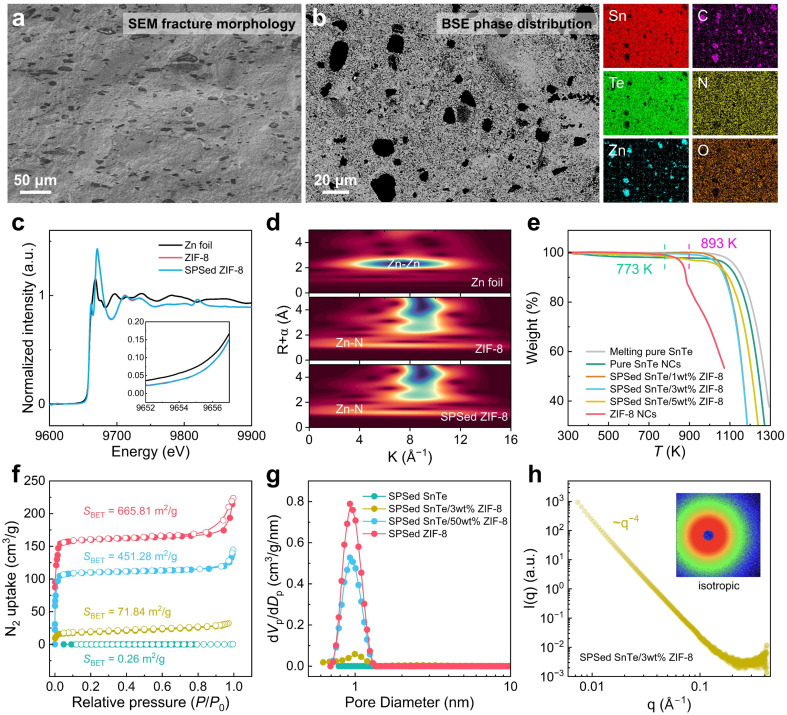
(a) SEM image of the fractured surface of as-SPSed SnTe/5wt%ZIF-8. (b) Back-scattered electron image of the polished surface of as-SPSed SnTe/5wt%ZIF-8 and corresponding EDS mapping of Sn, Te, Zn, C, N, and O elements. (c) Zn K-edge XANES spectra of Zn foil, ZIF-8, and as-SPSed ZIF-8. (d) Comparison of Zn K-edge EXAFS wavelet transform results of Zn foil, ZIF-8, and as-SPSed ZIF-8. (e) TG curves of the ZIF-8, pure SnTe, and as-SPSed SnTe/*x*wt%ZIF-8 (*x* = 1, 3, and 5) samples measured under a flow of N_2_. (f) 77 K nitrogen adsorption–desorption isotherms and (g) pore size distribution plots of as-SPSed samples SnTe, ZIF-8, SnTe/3wt%ZIF-8, and SnTe/50wt%ZIF-8. (h) SAXS scattering spectra of as-SPSed SnTe/3wt%ZIF-8 and the corresponding scattering pattern.

The SnTe/*x*wt%ZIF-8 (*x* = 0, 1, 2, 3, 4, and 5) nanocomposites were produced by ultrasonic stirring, followed by fast densification *via* spark plasma sintering (SPS). In addition to the noticeable SnTe matrix phase, we observed the secondary-phase peaks characteristic of ZIF-8 in the sintered nanocomposites (see Fig. 1g and S4). The intensity of these diffraction peaks increases gradually with increasing *x* content, showing the ZIF-8 phase with relatively high crystallinity in the SnTe/*x*wt%ZIF-8 (*x* = 0, 1, 2, 3, 4, and 5) system. No C peak is visible in any of the as-SPSed SnTe/ZIF-8 nanocomposites, which preliminarily demonstrates that the ZIF-8 phase can be stabilized more effectively in SnTe. We found that the peak positions of the SnTe phase did not shift upon increasing the ZIF-8 content from 0 to 5 wt%, indicating an absence of appreciable changes in the lattice parameters upon ZIF-8 addition. Moreover, no diffusive chemical fluctuation concerning the Zn element is observed at the SnTe/ZIF-8 heterointerface (Fig. S5). Therefore, the contribution of elemental doping to the SnTe matrix could be neglected. We also prepared a SnTe/50wt%ZIF-8 sample for investigating the large-scale survival of ZIF-8 in the SnTe matrix and the ZIF-8-composition-dependent evolution of the microstructure. As shown in Fig. S6, all the characteristic peaks of ZIF-8 are present in the Fourier transform infrared (FTIR) spectra of as-SPSed SnTe/50wt%ZIF-8 samples. These observations are in line with our design of SnTe/ZIF-8 hetero-composites (Fig. 1c). Cross-sectional SEM image of the pure SnTe sample presents coarse grains with an average size of around 4 µm (Fig. S7a), a typical feature of the effect of Ostwald ripening on grain size during the sintering process.^45^ Both the SEM fractography image (Fig. 2a and S8a) and backscattered electron (BSE) image (Fig. 2b and S8b) of SnTe/*x*wt%ZIF-8 (*x* = 3 and 5) samples reveal the existence of heterogeneous regions with different sizes, which are dispersedly distributed in the SnTe matrix. The corresponding EDS mapping results consistently display that the above regions are composed of ZIF-8, confirming that the dense dots around the matrix grain boundaries are fine ZIF-8 grains rather than other defects. The magnified cleavage surfaces provide a clear view of ultra-fine grain microstructures (<1 µm) in bulk SnTe/*x*wt%ZIF-8 (*x* = 3 and 5) (Fig. S7b and c), which is attributed to the Zener pinning effect^5,46,47^ induced *via* ZIF-8 micro/nanodomains. Specifically, ZIF-8 could inhibit the migration of SnTe grain boundaries (GBs) by decreasing the interfacial Gibbs free energy, resulting in suppression of the grain coarsening phenomenon under high temperature operating conditions.^7,48,49^

To examine the influence of high-temperature spark plasma sintering on the electronic and coordination structures of the ZIF-8 decoration layer, the X-ray absorption near-edge structure (XANES) and extended X-ray absorption fine structure (EXAFS) were used to analyze the representative pure and as-SPSed samples of ZIF-8, respectively. As shown in Fig. 2c, the absorption energy near the K edge of Zn^2+^ in the as-SPSed ZIF-8 was consistent with that of pure ZIF-8, indicating that as-SPSed ZIF-8 exhibited a strong interaction between Zn and N in 2-methylimidazole. Fourier-transform EXAFS spectra afforded short-range local structural information around Zn (Fig. S9). A prominent peak centered at 1.3 Å (uncorrected for phase shift) is due to nitrogen backscattering.^50^ This result is consistent with the Zn–N coordination in a typical ZIF-8 structure, and the absence of a Zn–O peak (2.9 Å) in the ZIF-8 sample indicates that the framework structure of ZIF-8 is effectively maintained throughout the SPS process. Wavelet transform (WT) analysis (Fig. 2d) also corroborated these observations, highlighting that the maximum intensity near 8.5 Å^−1^ for both ZIF-8 and as-SPSed ZIF-8 can be assigned to the Zn–N coordination. All the above experimental data (including XRD results in Fig. 1f) imply that the short- and long-range order of as-SPSed ZIF-8 is similar to that of well-crystallized ZIF-8.^51^

To further check the thermal instability temperature of the SnTe/ZIF-8 heterostructure, we then turned to the thermogravimetric (TG) technique to provide a quantitative thermolysis analysis on SnTe, ZIF-8, and the as-SPSed SnTe/*x*wt%ZIF-8 (*x* = 1, 3, and 5) composites (Fig. 2e). ZIF-8 thermolysis proceeds through a discernible two-stage dissociation, one at a lower *T* (773–893 K), and the other at a higher *T* (above 893 K), which are responsible for 7% (corresponding to one equivalent of methyl groups, which is why we limited the sintering range to the upper limit of 773 K) and complete decomposition of the ZIF-8 crystal, respectively. These values are coherent with results previously reported within the experimental uncertainty.^52,53^ No rapid weight loss was observed in both solvothermal and melting pure SnTe before the start of decomposition at around 1013 K (*i.e.*, the melting point of SnTe). In contrast, the as-SPSed SnTe/*x*wt%ZIF-8 (*x* = 1, 3, and 5) composite has a much more rapid mass loss (approximately 0.7%, 2.1%, and 3%, respectively) in the range of 893–1013 K, implying that the framework of the ZIF-8-layer starts to break apart at the Zn–N coordination bonds and the C–C bridging bonds between the imidazolate ligand and the methyl group. Compared to SnTe/3wt%ZIF-8 before SPS with a sharp weight loss starting from 773 K, the TG plot of SPSed SnTe/3wt%ZIF-8 remains largely unchanged until 893 K (Fig. S10). Thus, the minor mass loss observed above 893 K is attributed to the decomposition of ligand methyl groups, rather than the collapse of the ZIF-8 framework structure. Clearly, the ZIF-8 phase can be stabilized without the loss of overall framework connectivity from room temperature to 873 K through the creation of a heterostructure in bulk SnTe thermoelectrics.

The type-I N_2_ adsorption–desorption isotherms indicate the typical microporosity of as-SPSed ZIF-8, as-SPSed SnTe/3wt%ZIF-8, and as-SPSed SnTe/50wt%ZIF-8, except for as-SPSed SnTe (Fig. 2f). The Brunauer–Emmett–Teller (BET) specific surface area of the as-SPSed ZIF-8 is 665.81 m^2^ g^−1^, lower than the reported values for pristine ZIF-8 (1255–1600 m^2^ g^−1^).^54^ We attribute this reduction to the grain boundary stitching in ZIF-8, yielding some degree of locally disordered pore cavities after spark plasma sintering. Notably, SnTe/50wt%ZIF-8, SnTe/3wt%ZIF-8, and ZIF-8-free SnTe composites display a disproportionate decrease in BET surface area (451.28, 71.84, and 0.26 m^2^ g^−1^, respectively), suggesting the inheritance of ZIF-8's microporous structure. This can be ascribed to the enhanced thermal stability of ZIF-8 embedded in the SnTe matrix. It is thus credible that a significant fraction of the ZIF-8 porosity survives after the densification process even at low addition amounts (*e.g.*, 1, 2, 3, 4, and 5 wt%). The pore size distribution results indicate that the ZIF-8, SnTe/3wt%ZIF-8, and SnTe/50wt%ZIF-8 samples exhibit characteristic sub-nanopores centered around 9.5 Å (Fig. 2g). Fig. 2h and S11 show the small-angle X-ray scattering (SAXS) patterns of the SPSed SnTe/3wt%ZIF-8 pellet. The scattering intensities, *I*(*q*), were measured as a function of the scattering vector (*q*). This vector relates to the characteristic length scales (*d*) of the sample *via* the relationship *q* = 2π/*d*. The observed scattering arises from the electron density contrast between the SnTe matrix and the internal empty pores. The high *q* region (roughly *q* > 0.3 Å^−1^) corresponds to the features of micropores (pore diameter less than 20 Å), and the low *q* region (roughly *q* < 0.3 Å^−1^) corresponds to mesopores (pore diameter between 20 and 700 Å). The observation of the *q*^−4^ power law is commonly related to the sharp phase interface.

**Fig. 3:**
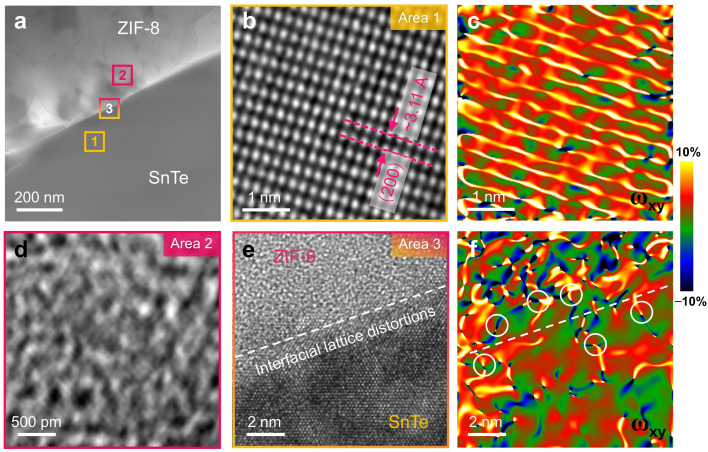
(a) Representative TEM image of the SnTe/ZIF-8 heterostructure. HAADF-STEM images of (b) SnTe, (d) ZIF-8, and (e) interfacial domain. In (c) and (f), the corresponding geometrical phase analysis (GPA) patterns calculated from the regions (b) and (e), respectively.

Fig. 3a shows a low-magnification transmission electron microscopy (TEM) image of a representative SnTe/ZIF-8 heterostructure, and its excellent bonding is confirmed by the clear and continuous interface morphology. We note that the ZIF-8 grain retains its initial size of ∼350 nm. To further investigate the atomic structure of SnTe, ZIF-8, and their interface, high-angle annular dark-field scanning transmission electron microscopy (HAADF-STEM) was performed. It is evident that SnTe (Fig. 3b) has a perfect lattice that reveals almost no visible defects. As shown in Fig. 3c, the strain field of the SnTe matrix is exhibited by the geometrical phase analysis (GPA) map, indicative of a relatively uniform internal lattice strain distribution. This is a manifestation of the poor impurity doping of the SnTe matrix, echoing well with the above-mentioned characterization results. The lattice striations of ZIF-8 were not obviously discernible (Fig. 3d), as ZIF-8, like most MOFs, is greatly sensitive to electron beam irradiation owing to its organic linker (2-methylimidazole) and porous framework. Under a high-energy electron beam, ZIF-8 tends to undergo rapid amorphization, making it highly difficult to witness evident lattice fringes. However, we remain able to find significant black microporosity in the bulk structure of ZIF-8. We have observed a highly fluctuating extension of atomic arrangements between SnTe and ZIF-8, as demonstrated in Fig. 3e. This verifies a high degree of lattice mismatching between these two phases, leading to a large interfacial lattice distortion (Fig. 3f). Such a strong spatially distributed strain would be responsible for strongly interrupting the phonon transport along different directions.

### 2.3 Electrical transport properties

**Fig. 4:**
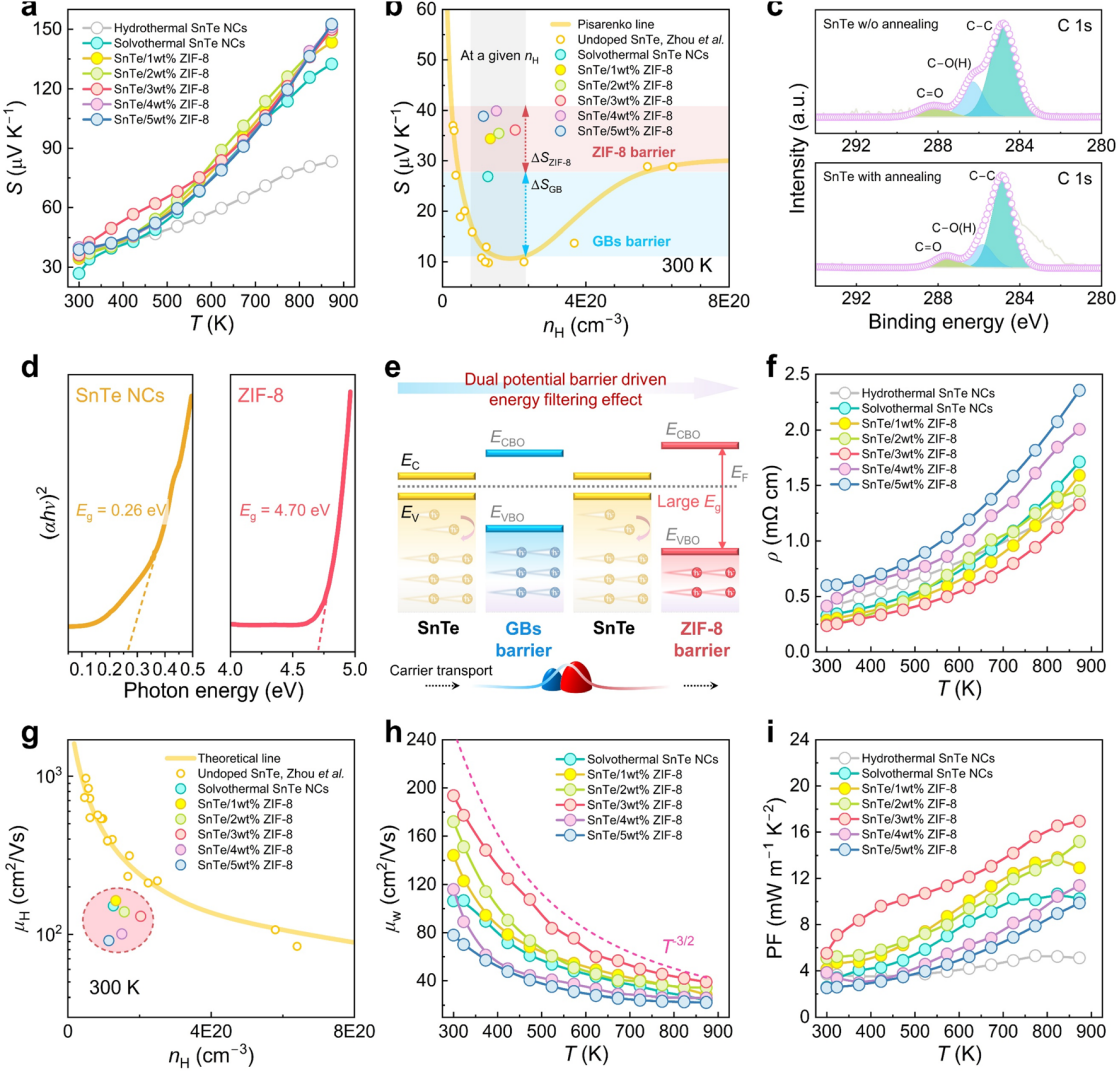
(a) Temperature-dependent Seebeck coefficient (*S*) for samples SnTe/*x*wt%ZIF-8 (*x* = 0, 1, 2, 3, 4, and 5), and hydrothermal SnTe for comparison. (b) Comparison of the Hall carrier concentration-dependent Seebeck coefficient at 300 K with literature data.^67^ The solid Pisarenko line is calculated based on the two-valence-band model, considering both light hole and heavy hole valence bands. The yellow circle is used to guide the eye. (c) High-resolution C 1s XPS spectra of solvothermal SnTe nanocrystals with and without annealing at 873 K for 3 h. (d) Optical bandgaps (*E*_g_) of SnTe and ZIF-8 NCs. The band gap values are determined from Tauc plots of the optical absorption coefficient (*αhν*)^1/*n*^*versus* photon energy *hν* (*h* indicates the Planck constant and *ν* represents the frequency of the photon), based on Fourier transform infrared (FTIR) measurements, where the value of *n* was taken to be 2 for direct-gap both SnTe^7^ and ZIF-8.^68^ (e) Schematic illustration of the interfacial band diagrams for SnTe/ZIF-8 nanocomposites, displaying the dual barrier (*i.e.*, GBs adsorbed with trace organics and ZIF-8 phases) driven energy filtering effect on hole carrier transport; *E*_C_, conduction band minimum; *E*_V_, valence band maximum; *E*_CBO_, conduction band offset; *E*_VBO_, valence band offset. Clearly, the *E*_VBO_ due to the large *E*_g_ discrepancy between SnTe and ZIF-8 is outside the tolerable range (within −0.4 to +0.3 eV) of valence band alignments. (f) Temperature-dependent electrical resistivity (*ρ*). (g) Comparison of the Hall carrier concentration-dependent Hall carrier mobility (*µ*_H_) at 300 K with those reported in the literature.^67^ Temperature-dependent (h) weighted carrier mobility (*µ*_w_) and (i) power factor (PF).

The electrical transport properties of all SnTe-based specimens were evaluated over the temperature range of 300 to 873 K. The solvothermal-synthesized Sn-rich SnTe nanomaterials in this work exhibit a significantly higher Seebeck coefficient (*S*) than those prepared by the hydrothermal route (Fig. 4a), as well as those obtained by other reported fabrication methods (high-temperature melting;^7,30,55^ liquid phase exfoliation;^56^ microwave-hydrothermal/solvothermal process^57–60^). The Sn-rich composition activates an intrinsic self-compensation mechanism,^61^ effectively suppressing the overly high Sn-vacancy density (*i.e.*, hole concentration) in SnTe. Consequently, the room-temperature Hall carrier concentration (*n*_H_) is tuned to a relatively low level of 1.25 × 10^20^ cm^−3^, more optimized than that of hydrothermal SnTe (∼9 × 10^20^ cm^−3^; Fig. S12a). However, the Sn self-compensation is still not enough to explain the markedly enhanced thermopower since a significant deviation in *S* can be seen in pristine SnTe as compared with the Pisarenko line at a given *n*_H_ (Fig. 4b; the blue region). Note that SnTe and PbTe employ identical chemical bonding mechanisms in which the half-filled p-band σ-bond (metavalent bonding) facilitates an octahedral rock-salt structure,^62^ leading to analogous “two-energy-band” structures. Here, the theoretical Pisarenko relationship between *n*_H_ and *S* at 300 K was supported by a two-valence-band model^7^ using the density of state (DOS) effective masses 
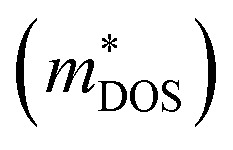
 of 0.168 *m*_e_ and 1.92 *m*_e_ (*m*_e_ being the unit mass of a free electron) for the L and Σ valence bands, respectively, and an energy separation (Δ*E*_L–Σ_) of 0.35 eV.^63^ Since the electronic band structure of SnTe should not change, given that the SnTe matrix was not doped, the density-of-states effective mass should also not change. Thus, the experimental data points above the theoretical Pisarenko line for SnTe should be induced by the interfacial energy filtering effect.^11^ It is well known that organic ligands are typically left on the solvothermally processed NC surface, unlike inorganic ligands, which may volatilize upon thermal consolidation or stabilization. Although the TG (Fig. 2e) plots of SnTe/*x*wt%ZIF-8 show negligible residual organic matter, the C 1s region of XPS spectroscopy reveals the conspicuous signs of C=O, C–O(H), and C–C bonds (Fig. 4c), indicating the presence of stable organic species on the surface of SnTe NCs regardless of high-temperature annealing treatment. Simultaneously, the high aging robustness of the transport properties shown in Fig. S13 proves that trace organics adsorbed onto SnTe have strong resistance to thermal decomposition. The exact species is not known, but it can be categorized as short organic groups, since DMF serves as a small-molecule solvent^64^ as well as a short-chain ligand for weak coordination in our solvothermal reaction. Such trace organics from the liquid-phase synthesis process are adsorbed on the nanostructures and turn into resistive GB phases between adjacent grains of bulk SnTe. In p-type SnTe, low-energy holes have a smaller contribution to the Seebeck coefficient (*S*). The energy barrier at the high-density resistive GBs would preferentially block these low-energy holes, leading to an increase in the interfacial and thus total thermopower. The detailed descriptions can be found in Discussion 2 of the SI. Given all that, the enhanced *S* in pure SnTe can be attributed to the *in situ* formation of Sn-rich stoichiometry and the GB energy barrier formed by the adsorption of trace organics on the NC surface. A similar phenomenon to the latter was also observed in other organic–inorganic nanocomposite thermoelectrics.^65,66^

After introducing ZIF-8, the *S* further rises in the full range of temperature for the nanocomposites with *x* = 1 to 5 compared to pristine SnTe. The room-temperature *S* value of 39.9 µV K^−1^ for the *x* = 4 sample is much higher than the 26.8 µV K^−1^ for ZIF-8-free SnTe (refer to *x* = 0) (Fig. 4b; the red region). A maximum *S* approaching 153 µV K^−1^ at 873 K is achieved for SnTe/5wt%ZIF-8 nanocomposites, without observable bipolar conduction. Although Zn doping in SnTe is theoretically known to be effective for converging light *L* and heavy *Σ* valence bands,^69^ experimentally, its solubility is found to be too low to enable a sufficient reduction in energy separation (Δ*E*_L−Σ_) of SnTe. Thereby, the impurity doping-induced band convergence effect on the bulk band structure and the related 
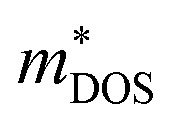
 can be neglected in this work. Considering the nearly constant *n*_H_ with increasing ZIF-8 addition, the increase in virtual 
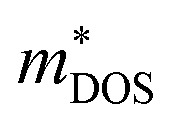
 on the Pisarenko curve could only stem from the energy filtering effect induced by the ZIF-8/SnTe interface. The room-temperature bandgap (*E*_g_) of ZIF-8 is measured to be 4.70 eV (Fig. 4d), which is strikingly larger than that of SnTe (0.26 eV), implying a band offset (*i.e.*, energy barrier) at the heterointerface. XPS results of the SnTe/3wt%ZIF-8 hetero-structured sample (Fig. S15–S17) validated the formation of Sn–N bonding because of complexation with the 2-methylimidazole ligand in ZIF-8. The metavalent bonding mechanism employed by SnTe led to multicenter and long-range interactions as well as large effective coordination numbers,^70,71^ so that foreign atoms could change the charge transfer from surrounding metavalent-bonded atoms,^35,72^ indicating the regulation of physical properties. From this perspective, such electronic rearrangement associated with Sn–N bonding could trigger the collapse of long-range metavalent bonds at SnTe/ZIF-8 interface regions, leading to breakdown of the dielectric screening and a larger number of trapping states.^10^ They would become space-charge regions upon trapping the free carriers and thus create potential energy barriers. In this work, we have constructed two types of interfacial energy barriers, the phase interfaces of matrix/resistive GBs and matrix/wide-band-gap-ZIF-8, in the nanocomposites. A potential barrier at a grain boundary would induce both the GB band offset (Δ*E*) and the interfacial resistance.^11^ Specifically, the band offset (Δ*E*) between the valence band maximum (VBM) of the grain and the GB can act as a hole carrier filter (Fig. S14). By changing the band structure at the GBs, the low-energy carriers that contribute less to *S* can be selectively “filtered out” (Fig. 4e). Moreover, we have increased the Kapitza thermal resistance of the GB phase by using both heterostructural organics and ZIF-8 as GB engineering additives. Kapitza resistance (*ρ*_Kapitza_) is a measure of the resistance of an interface to the transport of heat through it,^73^ commonly observed in composites due to differences in the physical properties of constituent materials and increases with increasing dissimilarity in phonon dispersion.^74^ This, in turn, would promote the temperature drop across the GB region, which further improves the interfacial *S* in SnTe. Ultimately, our SnTe/ZIF-8 nanocomposites are conducive to realizing enhanced total *S via* the dual-barrier-induced interfacial band offset and Kapitza resistance.

Fig. 4f plots the measured *T*-dependent *ρ* for our SnTe/*x*wt%ZIF-8 (*x* = 0, 1, 2, 3, 4, and 5) nanocomposites. The *ρ* of all the samples increases monotonically with increasing *T* up to 873 K, exhibiting a metallic transport behavior. Remarkably, the *ρ* values are nearly unchanged for three ZIF-8-containing nanocomposites (*x* = 1, 2, and 3), compared with that of the pure SnTe sample. Generally, ZIF-8 behaves as a poor electrical conductor owing to the lack of good π-conjugation pathways in the organic ligands,^19^ as well as the non-overlap between the p orbital and the d orbital of Zn^2+^.^20^ Thus, this is a direct manifestation of the good connection of the SnTe matrix and the resulting formation of a cross-linked network (Fig. 2a, b, S5 and S8). Meanwhile, the carrier-density-gradient-driven quantum tunneling effect^75^ (physically termed the penetration of matter waves and the transmission of particles through a high potential barrier) cannot be overlooked (Fig. S18), wherein the charge carriers could tunnel from a SnTe nanograin with a topological surface state (there is a close link between MVB and topological insulators)^2,76,77^ to another through a ZIF-8 semiconducting layer. For the cases with *x* = 4 and 5, markedly increased *ρ* values are observable at both ambient and elevated temperatures, which should arise from the overall *µ*_H_-reduction (Fig. S12b) deriving from carrier blocking/scattering *via* the macroscopic aggregation of porous ZIF-8. As shown in Fig. 4g, a slightly degraded room-temperature mobility is an inevitable consequence of porous ZIF-8 addition, where the holes with sub-nanoscale mean free paths (MFPs) may be collectively scattered. However, this effect appears to be relatively small compared to the prominent Sn-vacancy defect scattering in hydrothermal SnTe. In our solvothermal SnTe matrix, the limited Sn^0^ can fill vacancies under non-stoichiometric equilibrium and thus weaken inter-carrier scattering. More importantly, the scattered carriers are mainly low-energy charge carriers. To further evaluate the carrier transport characteristics upon heterointerface addition, the quantity of weighted carrier mobility (*µ*_w_), independent of the carrier concentration, was introduced in SnTe/*x*wt%ZIF-8 (*x* = 0, 1, 2, 3, 4, and 5) systems. Weighted mobility (*µ*_w_) is an experimental material parameter that can be computed from the measured values of the Seebeck coefficient (*S*) and electrical conductivity (*σ* = 1/*ρ*).^78^ The temperature-dependent weighted carrier mobility (*µ*_w_) follows a power law of *T*^−3/2^ over the entire measured temperature range (Fig. 4h), suggesting that the acoustic-phonon deformation potential scattering mechanism dominates the charge transport. The enhanced thermopower *S* due to the low-energy carrier scattering and well-maintained *µ*_H_ due to the cross-linked SnTe network (see Fig. S5), contribute to large *µ*_w_ that supports the enhanced power factor (PF) in the SnTe/ZIF-8 system with a low amount of ZIF-8 addition. The SnTe/3wt%ZIF-8 achieves a maximum PF of *ca.* 16.93 µW cm^−1^ K^−2^ at 873 K while the ZIF-8-free SnTe shows a value of 10.24 µW cm^−1^ K^−2^ (Fig. 4i). This value is comparable to those of other state-of-the-art SnTe thermoelectrics synthesized by the solution-phase method.^5,32,57,79–83^

### 2.4 Thermal transport properties

**Fig. 5:**
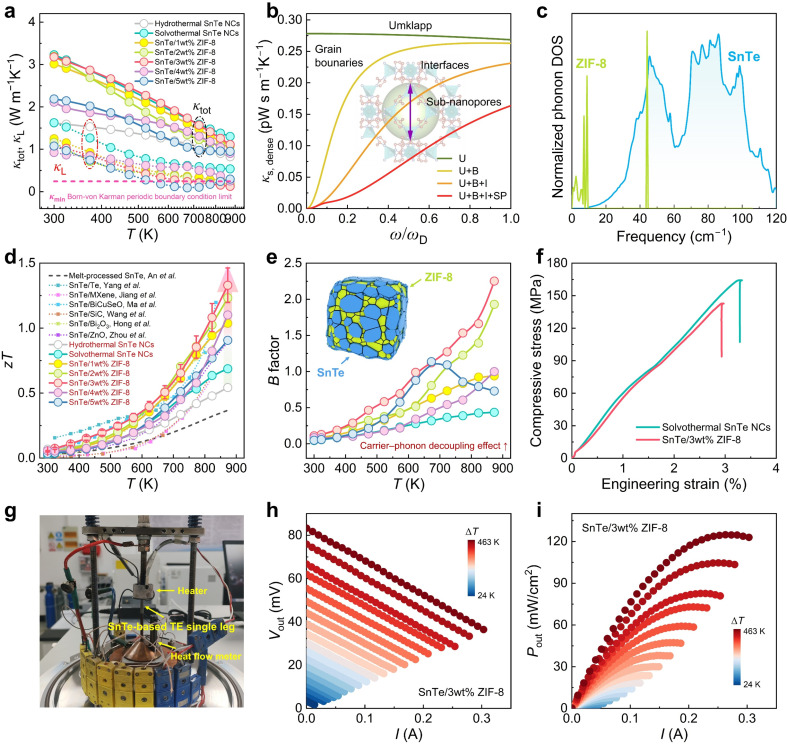
(a) Temperature-dependent total and lattice thermal conductivity (*κ*_tot_ and *κ*_L_). (b) The calculated spectral thermal conductivity (*κ*_s, dense_) for SnTe/3wt%ZIF-8 using the modified Debye–Callaway model with various phonon scattering mechanisms at 300 K. (c) Phonon DOS of the SnTe and ZIF-8 phase. (d) Temperature-dependent figure of merit (*zT*) of hydrothermal SnTe and SnTe/*x*wt%ZIF-8 (*x* = 0, 1, 2, 3, 4, and 5) pellets in comparison with other hetero-structured SnTe thermoelectrics.^96–101^ (e) Temperature-dependent quality factor *B*. (f) Compressive stress and strain curves of solvothermal SnTe and SnTe/3wt%ZIF-8. (g) Photograph of the single-leg thermoelectric device. Current (*I*)-dependent (h) output voltage (*V*_out_) and (i) output power density (*P*_out_) for SnTe/3wt%ZIF-8.

The temperature dependences of the total and lattice thermal conductivities (*κ*_tot_ and *κ*_L_) for the SnTe/*x*wt%ZIF-8 (*x* = 0, 1, 2, 3, 4, and 5) nanocomposite are displayed in Fig. 5a, where *κ*_L_ is calculated by subtracting the electronic thermal conductivity (*κ*_e_) from *κ*_tot_ based on the Wiedemann–Franz relationship (*κ*_e_ = *LT*/*ρ*). The Lorenz number *L* is calculated using the equation *L* = 1.5 + exp(−|*S*|/116) × 10^−8^ V^2^ K^−2^.^84^ It is found that *κ*_tot_ gradually diminished with increasing *x* content over the full range of temperature. The SnTe/5wt%ZIF-8 sample exhibits the lowest *κ*_tot_, decreasing from ∼2.18 at 300 K to ∼0.95 W m^−1^ K^−1^ at 873 K, with increasing temperature. For the lattice contribution, *κ*_L_ essentially follows a monotonically declining tendency with increasing ZIF-8 content, where the room-temperature value decreases by ∼43% from 1.62 W m^−1^ K^−1^ for the *x* = 0 sample to 0.92 W m^−1^ K^−1^ for the *x* = 4 sample. At high temperatures (for example, 873 K), phonon blocking by the incorporation of ZIF-8 is more effective, ensuring a remarkable reduction (∼76%) from 0.54 W m^−1^ K^−1^ for the intrinsic sample of SnTe to 0.18 W m^−1^ K^−1^ for the *x* = 3 sample. The latter value is lower than the theoretical lower bound *κ*_L, min_ ∼0.24 W m^−1^ K^−1^ estimated by a diffuson-mediated transport model and the one that considers the periodic boundary conditions (Born–von Karman approximation).^38^ Note that an overestimation of the predicted *κ*_L, min_ may be present due to the lack of the rationalized phonon dispersion for SnTe solid with imperfections.^85^ Importantly, it is one of the experimentally determined lowest values compared with the literature data for heavily doped SnTe-based thermoelectrics, such as ∼0.26 W m^−1^ K^−1^ at 900 K in (SnTe)_0.92_(MnCd_0.6_Ge_0.4_Te_2_)_0.08_,^39^ ∼0.25 W m^−1^ K^−1^ at 823 K in (Sn_0.79_Sb_0.14_)_0.8_Ge_0.2_Te_0.8_Se_0.2_,^40^ ∼0.32 W m^−1^ K^−1^ at 873 K in Sn_0.7_Ge_0.2_Mn_0.08_In_0.02_,^41^ ∼0.32 W m^−1^ K^−1^ at 773 K in (SnTe)_0.8_(Ag_1.05_PbSb_0.95_Te_3_)_0.2_,^8^ ∼0.36 W m^−1^ K^−1^ at 873 K in SnAg_0.05_Te–10%CdSe,^7^ ∼0.35 W m^−1^ K^−1^ at 873 K in Sn_0.79_Ge_0.15_Sb_0.06_Te–4%CdTe,^6^ and ∼0.28 W m^−1^ K^−1^ at 865 K in SnTe–Ag/Mn.^42^ These results highlight that a sufficiently reduced *κ*_L_ can be achieved using interfacial heterogeneity without alloying the matrix lattice.

Next, we investigate the effect of the obtained microstructure on the ultralow lattice thermal conductivity. We first use a classical effective medium theory (EMT) model developed by Bruggeman and Landauer^86,87^ to analyze the different lattice-defect contributions to the suppression of *κ*_L_, involving the porosity-induced thermal radiation and multiscale phonon scattering. Since the EMT function *f*_*κ*_(*ε*) depends weakly on the pore shape,^88^ the net contribution of thermal radiation to the *κ*_L_-reduction can be described by *κ*_L,R_ = *f*_*κ*_(*ε*)*κ*_L, dense_ = (1–3*ε*/2)*κ*_L, dense_,^89^ where *κ*_L, dense_ is the *κ*_L_ of the imaginary fully dense material and *ε* is the void fraction. Notably, the absence of a medium in the pores would proportionally decrease the thermal conductivity (*κ*) and the electrical conductivity (1/*ρ*), causing no net gain in the *zT* value. Fortunately, ZIF-8 forms closed pores embedded in the GBs of incipient metal SnTe, which results in the intense pore–interface scattering of phonons, while simultaneously maintaining good charge transport between matrix grains. On the other hand, the fully dense nanocomposite *κ*_L, dense_ is calculated using a modified Debye–Callaway model,^90,91^ wherein *κ*_L, dense_ is the integral of the spectral lattice thermal conductivity (*κ*_s, dense_) with respect to the phonon frequency (*ω*):1
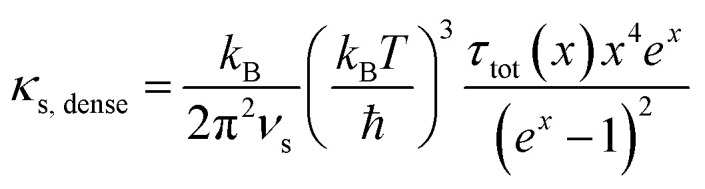
where *k*_B_, *ħ*, *ν*_s_, *τ*_tot_, and *x* are the Boltzmann constant, reduced Planck's constant, average sound velocity, total phonon relaxation time, and reduced frequency (defined as *ħω*/*k*_B_*T*), respectively. Based on the above microstructure characterization, *τ*_tot_ can be ascribed to four phonon scattering modes resulting from the Umklapp processes (U), grain boundaries (B), SnTe/ZIF-8 heterostructure interfaces (I), and sub-nano pores (SP) according to Matthiessen's law: 
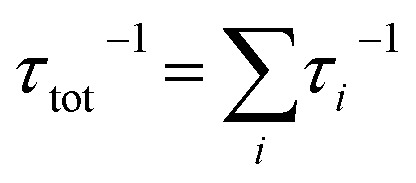
. The detailed calculations can be found in the SI. The modelling parameters were determined from various experiments (Table S1). Fig. 5b displays the plots of calculated *κ*_s, dense_*versus* the normalized phonon frequency *ω*/*ω*_D_ (*ω*_D_ denotes the Debye frequency) for SnTe/3wt%ZIF-8, where the area between the two plots corresponds to the amount of *κ*_L_-reduction from the specified scattering mechanism. As can be seen, the inherent U scattering causes a relaxation of *τ*_U_ ∼ *ω*^−2^, effectively scattering full-*ω* phonons; the B scattering enabled by well-maintained nanograins plays a vital role in phonon scattering at low-*ω*; SnTe/ZIF-8 heterointerfaces trigger strong I scattering focused on mass fluctuation and thus effective phonon scattering in the medium-*ω* range; smaller sub-nanopores (∼9.5 Å in diameter) impede shorter mean free path (MFP) phonons and lead to a sharper decline in *κ*_L_ in the high-*ω* spectrum. As a result, such a defect configuration can account for the suppression of phonon propagation at both low and high temperatures. Nevertheless, the experimental *κ*_L_ values of SnTe/3wt%ZIF-8 samples at both 300 K and 873 K are still lower than the predicted values according to the EMT model (see Table S2), implying additional phonon blocking mechanisms.

In fact, the SnTe/ZIF-8 nanocomposites studied here are complicated, comprising both homogeneous grains and series-connected heterogeneous interface/boundary regions, which is one of the limiting cases of effective medium theory (EMT).^92,93^ In this case, the interfacial thermal resistance (*i.e.*, Kapitza resistance *ρ*_Kapitza_^73^) should also constitute an impediment for phonon transport in the SnTe/*x*wt%ZIF-8 system. From the viewpoint of acoustic and/or diffuse mismatch models,^74,94^ the structural difference across a two-dimensional solid/solid interface could induce an effective phonon scattering interfacial region with several nanometres in thickness. We carried out DFT calculations for the phonon density of states (PDOS) of SnTe and ZIF-8 using supercell models (Fig. S19) of Sn_32_Te_32_ and 2 × 2 × 2 ZIF-8, respectively. SnTe has long been considered a metavalent solid that features soft Sn–Te chemical bonding and strong lattice anharmonicity,^2,70^ engendering a broad dispersion of phonon DOS between 0 and 120 cm^−1^ (Fig. 5c). In contrast, ZIF-8 exhibits a much more localized and lower phonon frequency. This is due to the fact that the configuration of ZIF-8 lies between crystalline solids and amorphous polymers; the thermal transport thus has two channels, *i.e.*, crystal lattice modes and localized molecular modes. On the one hand, Zn atoms of the ZIF-8 framework are mainly located at 0–8 cm^−1^.^95^ On the other hand, the functional groups (*e.g.*, CH_3_) with heavy atomic masses possess much lower vibration frequencies. The hybridization of these modes could reduce vibrational lifetimes and also promote an increase in localized incoherent modes.^22^ More importantly, the DOS maps reveal that the overlap between SnTe and ZIF-8 is very limited, with the overlapping frequency ranges confined to 0–10 and 42–43 cm^−1^. Such poor phonon coupling between these two materials would contribute to lower thermal conductivity at the interface. From the viewpoint of chemical bonding, different bonding mechanisms in the ZIF-8 and SnTe matrix could achieve a strong phonon dispersion mismatch across the hetero-interface. The phonon dispersion depends on the force constant *K* and atom mass *M* in the form of 
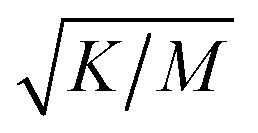
.^7^ The mismatch in interfacial chemical bonding could induce the interfacial strain field distribution for the suppression of phonon propagation (as shown in Fig. 3f). Accordingly, the overall thermal resistance of our SnTe/ZIF-8 system can be considered as the sum of thermal resistance in the grain phase and Kapitza resistance (*ρ*_Kapitza_) at the grain boundary.^11^ The inclusion of high-density nano-ZIF-8 in the grain boundary of SnTe nanomaterials increases Kapitza resistance, which ultimately enables a drastic *κ*_L_-decline in bulk SnTe/ZIF-8 nanocomposites, with the help of porosity-induced thermal radiation and multiscale phonon scattering in the SnTe grain phase.

### 2.5 *zT* values and device performance

The temperature dependences of the figure of merit (*zT*) values of SnTe/*x*wt%ZIF-8 (*x* = 0, 1, 2, 3, 4, and 5) nanocomposites are presented in Fig. 5d. For the case of pristine SnTe, a maximum *zT* of ∼0.69 at 873 K is obtained in the *x* = 0 sample, exhibiting an improvement of ∼92% and ∼28% in comparison with that of melt-processed and hydrothermal-processed SnTe, respectively. Attributable to the improved power factor caused by Sn self-compensation and the dual-barrier energy filtering effect, as well as suppressed *κ*_tot_ originating from the high-density sub-nano-porous ZIF-8/SnTe heterostructures, the thermoelectric properties of the SnTe/*x*wt%ZIF-8 system were significantly enhanced, especially yielding a peak *zT* of ∼1.3 at 873 K in SnTe/3wt%ZIF-8. This result dramatically surpasses those of other heterostructure-enhanced SnTe thermoelectric materials (such as SnTe/ZnO,^96^ SnTe/Te,^97^ SnTe/SiC,^98^ SnTe/MXene,^99^ SnTe/BiCuSeO,^100^ and SnTe/Bi_2_O_3_ (ref. 101)). The consistent *S*, *ρ*, *κ*_tot_, and *zT* of the SnTe/3wt%ZIF-8 sample upon repeated heating and cooling cycles corroborate its excellent thermal stability and reproducibility (Fig. S20). To gain a deeper understanding of the carrier–phonon decoupling effect, the composition dependence of the quality factor *B* at different temperatures was introduced, as shown in Fig. 5e. The *B* factor is determined based on an effective mass model:^78^2
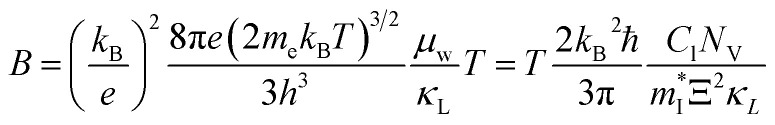
where *N*_V_ denotes the degeneracy of the band, *C*_l_ represents the average longitudinal elastic constant, 
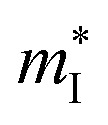
 signifies the inertial effective mass, and *Ξ* stands for the deformation potential coefficient (characterizing electron–phonon coupling strength). The *B* factor is proportional to the ratio of *µ*_w_ to *κ*_L_. The *B* factor achieves significant enhancement with increasing *x* (excluding *x* = 5), especially in the high-temperature range. The *B* values at 873 K for pure SnTe, *x* = 1, *x* = 2, and *x* = 3 samples are 0.43, 0.93, 1.92, and 2.25, respectively, indicating that our interface architecture scatters phonons while simultaneously enabling carrier transport. The interfacial ZIF-8 decoration engineering realized the synergistic optimization of electrical and thermal transporting properties of the SnTe system.

Thermoelectric materials should also have favorable mechanical stability for the next production of devices. As shown in Fig. 5f, the *x* = 3 sample shows a similar room-temperature compressive strength (142 MPa) to the pristine one (164 MPa), comparable to most of the reported values for state-of-the-art SnTe thermoelectric materials.^8,102–105^ Finally, we prepared a single-leg device and tested its thermoelectric conversion performance at different temperature gradients (Fig. 5g). As shown in Fig. 5h, the output voltage (*V*_out_) decreases linearly with increasing current (*I*). The *V*_out_ increases from 2 mV (Δ*T* = 24 K) to 83 mV (Δ*T* = 463 K) with increasing temperature. The variations in output power density (*P*_out_) with current (*I*) at different temperature differences (Δ*T*) are shown in Fig. 5i. It was found that *P*_out_ steadily increases as *I* increases, peaking at a specific Δ*T* while the external electrical load matches the internal resistance of the device. A maximum of *P*_out_ of ∼130 mW cm^−2^ was achieved at a hot-side temperature (*T*_h_) of 800 K and a temperature difference (Δ*T*) of 463 K, displaying promising potential for high-temperature heat harvesting. Further research on thermoelectric device geometry optimization based on the thermal-matching rule is expected to markedly improve its performance.

## 3 Conclusions and outlook

Despite the efforts devoted to developing solid-state porous materials associated with metal–organic frameworks (MOFs) for thermoelectric conversion applications, the gap between the overall thermal decomposition temperature of MOFs and the need for a highly thermostable phonon-glass electron crystal (PGEC) system capable of meeting the high-temperature power generation requirements remains a grand challenge. In this work, we present a successful example of a SnTe-based thermoelectric nanomaterial decorated with sub-nanoporous ZIF-8 layers, which exhibits both high thermal stability (>773 K) and a record thermoelectric figure of merit (*zT* ∼1.3 at 873 K) in this class of hetero-structured SnTe materials without doping the matrix lattice. Two reasonable explanations for exceptional resistance to thermal decomposition of SnTe/ZIF-8 hetero-structures can be advanced: first, internally in ZIF-8, bonding between imidazolate and Zn^2+^ is among the most stable for N-donor ligands^106^ at the level of metal-complex formation constants. This can stem from the existence of ionicity (the ligands in ZIF-8 are anionic) and partial covalency in the Zn–N bond; and second, externally, Zn possesses extremely low solubility in SnTe, preventing a pathway for thermal diffusion, further resulting in node stability. In combination, these two features of SnTe/ZIF-8 impart thermal stability more akin to that of covalent solids. In terms of thermoelectric response, we discovered that the *in situ* synthesis of tin-rich SnTe nanocrystals is responsible for optimized *n*_H_ in all the SnTe/ZIF-8 composites. The cross-linked network of the SnTe matrix allows for well-maintained carrier mobility in the low-ZIF-8-content samples. Given the significant difference between the bandgaps of ZIF-8 and SnTe, as well as the trace adsorption of organic species on the NC surface, the ZIF-8/SnTe heterointerface induces a dual-barrier-driven energy filtering effect, causing an increase in the interfacial *S* and thus the total *S,* and further enhancement of the electrical power factor. Additionally, effective phonon blocking is achieved by utilizing the abundant voids and heterogeneity of atomic masses and stiffness of bonds in a well-retained porous ZIF-8 decoration layer and constructing phase boundaries with considerable interfacial thermal resistance. This unique defect architecture produces full-spectrum phonon scattering leading to an ultralow *κ*_L_ = 0.18 W m^−1^ K^−1^ at 873 K. The representative SnTe/3wt%ZIF-8 sample exhibits satisfactory reproducibility and mechanical stability.

In this work, the combination of a zinc-based zeolitic imidazolate framework (ZIF-8) and p-type SnTe semiconducting nanomaterials offers a novel paradigm to overcome the stability bottlenecks of MOF-enhanced high-temperature thermoelectrics. The structural and performance tunability of MOFs can be achieved through synthesis strategies such as ligand functionalization, metal ion replacement, and pore size regulation. Thermally, the dispersed channels of MOFs enable precise matching with phonons of various wavelengths, facilitating efficient scattering of broad-spectrum phonons and providing a structural basis for the limiting suppression of *κ* at high *T*. Electronically, their metal–organic synergistic compositions endow the materials with unique electronic structures, where the charge transfer characteristics of metal ions and the potential π-electron conjugated systems of specific organic ligands collaboratively modulate carrier transport, creating conditions for the balanced optimization of *ρ* and *S*. More importantly, these rich tunability tools allow the construction of stable MOF-containing composites suitable for diverse temperature ranges and distinct semiconductor systems through molecular design and synthesis regulation to ensure directional optimization of the three key elements of thermoelectricity. Looking ahead, thermally stable MOF materials like ZIF-8, characterized by distinct structural features and controllable properties, are expected to serve as universal functional components, widely integrated into various thermoelectric systems (from room *T* to medium- and high-*T* thermoelectric solids). Through the deep integration of structural design and performance regulation, the decoupling limitations of traditional thermoelectric materials (such as Bi_2_Te_3_, PbTe, SnSe, and GeTe) could be overcome, paving the way for new avenues in the development of high-performance thermoelectrics.

## Author contributions

D. An, Y. Yu, and X.-M. Zhang conceived the research. W. Yue prepared the samples and conducted the thermoelectric property measurements. D. An carried out the SAXS experiments. J. Ren, F. Chen and Z. Hao collected and analyzed the (S)TEM data. J. Song, Z. Li and W. Yang performed the EXAFS characterization studies. D. An, W. Yue, and Y. Yu wrote the manuscript. S. Chen and X.-M. Zhang revised the manuscript. All the authors provided useful discussions and commented on the manuscript.

## Conflicts of interest

The authors declare no competing interests.

## Supplementary Material

SC-OLF-D6SC04137J-s001

## Data Availability

The data supporting this article have been included as part of the supplementary information (SI). Supplementary information is available. See DOI: https://doi.org/10.1039/d6sc04137j.
