# Supplementary material for: Stabilizing sub-nanoporous zinc metal–organic frameworks in SnTe thermoelectrics for high-temperature power generation
Source: Chem Sci. 2026 Jun 22. Online ahead of print. doi: 10.1039/d6sc04137j (PMC13285286; doi:10.1039/d6sc04137j)
Supplement: SC-OLF-D6SC04137J-s001 [file SC-OLF-D6SC04137J-s001.pdf]

Supplementary Information (SI) for Chemical Science.
This journal is © The Royal Society of Chemistry 2026

Supporting Information

Stabilizing sub-nanoporous zinc metal–organic frameworks in SnTe thermoelectrics for high-temperature power generation

Wang Yue[1†], Decheng An[1*†], Jiali Ren[1], Fangyuan Chen[1], Zhijun Hao[1], Jiaxi Song[1], Zejin Li[1], Wutao Yang[1], Shaoping Chen[1], Yuan Yu[2*], and Xian-Ming Zhang[1*]

[1] College of Chemistry and Chemical Engineering, State Key Laboratory of Clean and Efficient Coal Utilization, Analysis Testing and Equipment Sharing Center, Taiyuan University of Technology, Taiyuan 030024, China

[2] Institute of Physics (IA), RWTH Aachen University, Sommerfeldstraße 14, 52074 Aachen, Germany

[*]Corresponding author: andecheng@tyut.edu.cn (D. A.); yu@physik.rwth-aachen.de (Y. Y.); zhangxianming@tyut.edu.cn (X.-M. Z.)

[†] Equally contributed to this work

Methods

Materials

Tellurium powder (Te, 99.99%), tin (II) chloride anhydrous ($SnCl_2$, 99.99%), sodium hydroxide (NaOH, 99.99%), sodium borohydride ($NaBH_4$, 98%), N,N-dimethylformamide (DMF, 99.9%), zinc nitrate hexahydrate ($Zn(NO_3)_2{\cdot}6H_2O$, 99%), 2-methylimidazole (MeIM, 98%), methanol (CH_3OH, 99.5%), and absolute ethanol (C_2H_5OH, 99.9%) were supplied by Shanghai Aladdin Biochemical Technology Co., Ltd. All chemicals and reagents were used as received without further purification.

Synthesis of SnTe nanocrystals

SnTe nanocrystals (NCs) were synthesized via a facile solvothermal route. In a typical synthesis, Te (1.276 g) and $SnCl_2$ (2.257 g) were added to a mixed solution of DMF and NaOH, followed by the addition of sufficient sodium borohydride (1.893 g) to reduce Te and Sn^{2+}. The resulting suspension was stirred for 30 min at room temperature and then sonicated for 30 min to form a uniform suspension. The mixture was transferred into a 500 mL Teflon-lined stainless-steel autoclave, sealed and heated at 180 °C, for 20 h, and then naturally cooled down to room temperature. The obtained products were collected by centrifuging and washed with deionized water and absolute ethanol at least three times, and then were vacuum dried at 60 °C for 12 h.

Synthesis of ZIF-8 nanocrystals

In a normal procedure, $Zn(NO_3)_2{\cdot}6H_2O$ (1.12 g) was dissolved in 30 mL of methanol; then 2-methylimidazole (1.24 g) in 30 mL of methanol was subsequently added to the above solution under continuous stirring for 10 min and then via ultrasonic treatment for 10 min. After that, the suspended liquid was transferred to a Teflon-lined stainless-steel autoclave (50 mL) and heated at 120 °C for 2 h in an oven. After cooling to room temperature, the product was isolated from the filtrate as a white powder following centrifugation at 10000 rpm for 3 min, and then washed three times with methanol. Finally, the products were obtained by vacuum drying for 12 h at 60 °C.

Fabrication of SnTe/ZIF-8 nanocomposites

For the synthesis of 3 g of SnTe/*x*wt%ZIF-8 (x = 0, 1, 2, 3, 4, 5, and 50) samples, ZIF-8 powders (0 g, 0.03 g, 0.06 g, 0.09 g, 0.12 g, 0.15 g and 1.5 g) were firstly ultrasound

dispersed into ethanol, and SnTe powders (3 g, 2.97 g, 2.94 g, 2.91 g, 2.88 g, 2.85 g and 1.5 g) were then added into the ZIF-8 solution and ultrasonic together until evenly dispersed. After a period of intense sonication and stirring, SnTe/ZIF-8 nanocomposite solutions were formed. The as-obtained precipitates were centrifuged and dried in a vacuum at 60 °C overnight. For the preparation of bulk materials, the as-prepared powders were consolidated into ~2 mm thick, 13.5 mm diameter discs using spark plasma sintering (SPS-211Lx, Japan) at 773 K for 5 min in a cylindrical graphite mold at an axial pressure of ~45 MPa under ultrahigh vacuum.

Material characterizations

X-ray diffraction (XRD) patterns were characterized by SmartLab SE X-ray diffractometer (Rigaku, Japan) equipped with a Cu K_α source (λ = 1.5418 Å). The surface morphologies and structures of the samples were characterized using scanning electron microscopy (SEM) equipped with energy-dispersive X-ray spectroscopy (EDS). X-ray photoelectron spectra (XPS) were recorded on an X-ray photoelectron spectrometer (ESCALAB Xi+, Thermo Scientific, USA). (Scanning) Transmission electron microscopy (TEM/STEM) observations were conducted in a FEI Themis Z. STEM foils were prepared by a conventional procedure including cutting, grinding, dimpling, polishing, and Ar ion-milling (Gatan PIPS Model691). Optical absorption spectra were measured at 300 K based on a Bruker Optik GmbH INVENIO R FTIR spectrophotometer (k = 4000−400 cm^{-1}) equipped with an integrated sphere and a Perkin-Elmer Lambda 850+ UV-vis spectrophotometer (λ = 800−250 nm), respectively. Thermogravimetric analysis was carried out on a thermogravimetric analyzer (STA8122, Rigaku, Japan) in the temperature range 25−1000 °C under N_2 atmosphere. The specific surface areas of the samples were measured by a Quantachrome Autosorb-1 surface area and porosity analyzer at 77 K and using N_2 as the detection gas. Note that the powder samples for BET adsorption-desorption measurement were hand-ground from the spark plasma sintered ZIF-8, SnTe/3wt%ZIF-8, SnTe/50wt%ZIF-8, and SnTe bulk pellets. The grinding time of all samples was kept consistent (10 min) to enable adequate exposure of fresh grain boundaries (GBs). Zn K-edge X-ray absorption near-edge spectroscopy (XANES) and extended X-ray absorption fine structure (EXAFS) data were measured using a bench-top

easy XAFS300+ instrument (EasyXAFS LLC). The spectra were acquired using a Si 551 spherically bent crystal analyzer and an Ag anode X-ray tube, respectively. The spectra were dead-time corrected and the energy was calibrated using a Zn foil standard. The compressive mechanical properties of SnTe-based samples (3×3×6 mm) at room temperature were assessed using a universal testing machine equipped with a crosshead displacement rate of 0.1 mm/min. A 5 m Xenocs Xeuss 2.0 small-angle X-ray scattering SAXS equipped with dual microfocus (copper/molybdenum) sources and a Pilatus 3R 300 K hybrid photon counting detector was used. Using this equipment, scattering data were collected for the sample SnTe/3wt%ZIF-8. All FTIR spectra of the as-prepared samples were recorded on a Thermo Nicolet iS50 spectrometer in a range of 400−4000 cm^{-1} at a resolution of 4 cm^{-1}.

Thermoelectric properties measurements

The measurement uncertainty of S, σ, and κ_{tot} is about 5%, 5%, and 7%, respectively. The electrical transport properties, including electrical resistivity (ρ), Hall coefficient (R_H) and Seebeck coefficient (S), were measured under a helium atmosphere in the temperature range from 300 K to 873 K. The Seebeck coefficient was measured from the slope of the thermopower versus temperature differences within 0−5 K. The resistivity and Hall coefficient were measured using the van der Pauw technique under a reversible magnetic field of 2.0 T. The Hall carrier concentration (n_H) was determined by $n_H = 1/(eR_H)$. The thermal diffusivity (D) was measured using a laser flash technique (Netzsch LFA467) and the total thermal conductivity (κ_{tot}) was calculated $\kappa_{tot} = D\rho C_p$, where the density (ρ) of the sample was determined using Archimedes principle. Specific heat capacity (C_p) is the heat capacity determined by the Dulong–Petit limit and is assumed to be temperature independent. The output power (P_{out}) and output voltage (V_{out}) of SnTe/3wt%ZIF-8 single-leg device under varying temperature gradients were measured in a vacuum atmosphere.

Density-functional-theory calculation

We apply first-principles calculations according to density functional theory (DFT), implemented in the PWmat package using GPU[1, 2]. In detail, the calculation of phonon properties of two structures is realized by PWmat and the tool phonopy[3]. For the

exchange-correlation potential, generalized gradient approximations (GGA)[4] of the Perdew-Burke-Ernzerhof (PBE) functional[5] is adopted and used in the geometry optimization with the force tolerance for the maximal residual force of 0.001 eV/Å as the convergence criteria. The self-consistent calculations apply a convergence energy threshold of 10^{-8} eV. The Monkhorst-Pack *k*-points meshes of 2×2×2 for geometry optimization. Norm-Conserving Pseudopotential[5] with a cutoff energy of 70 Rydberg has been used for all the calculations in the PWmat package.

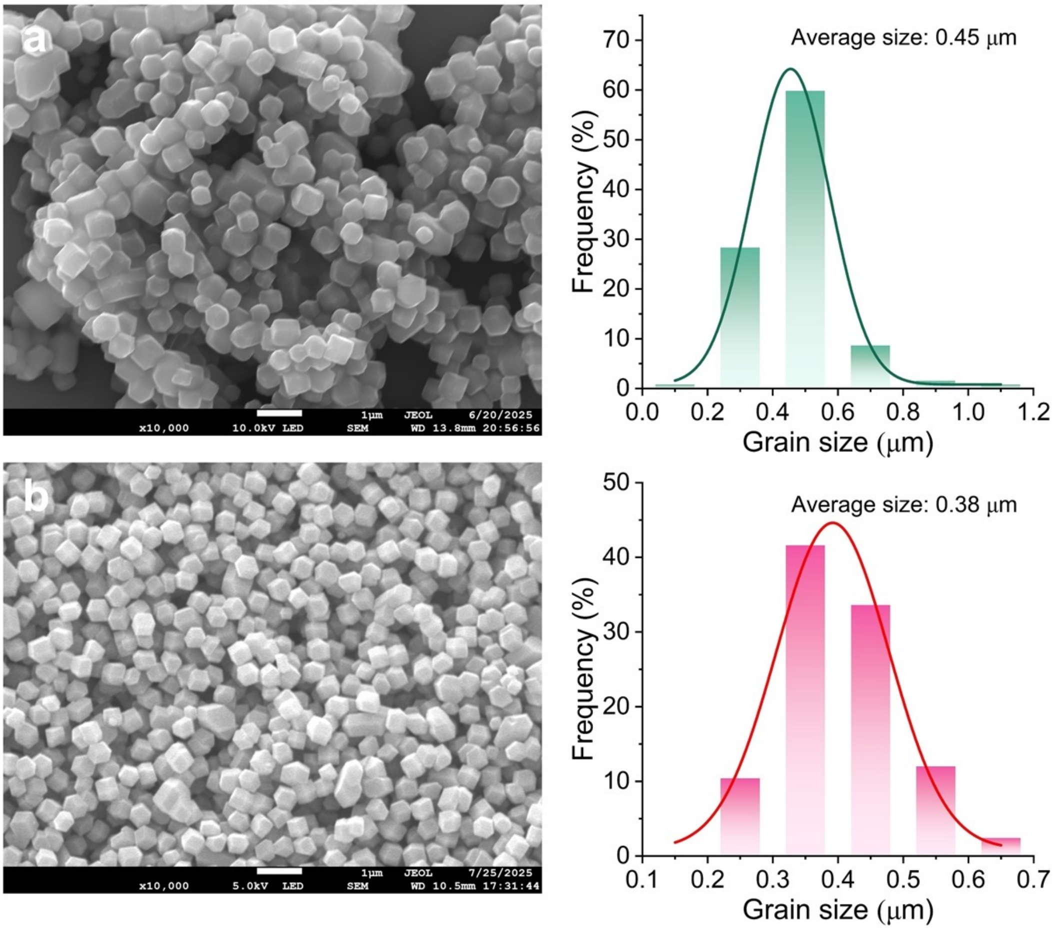

Figure S1. SEM morphologies and grain size distributions for (a) SnTe NCs and (b) ZIF-8 NCs. SEM was used to determine nanocrystal diameter by averaging the length of ~120 crystals per sample using Fiji, ImageJ. From the analysis, nanocrystal sizes were averaged was calculated.

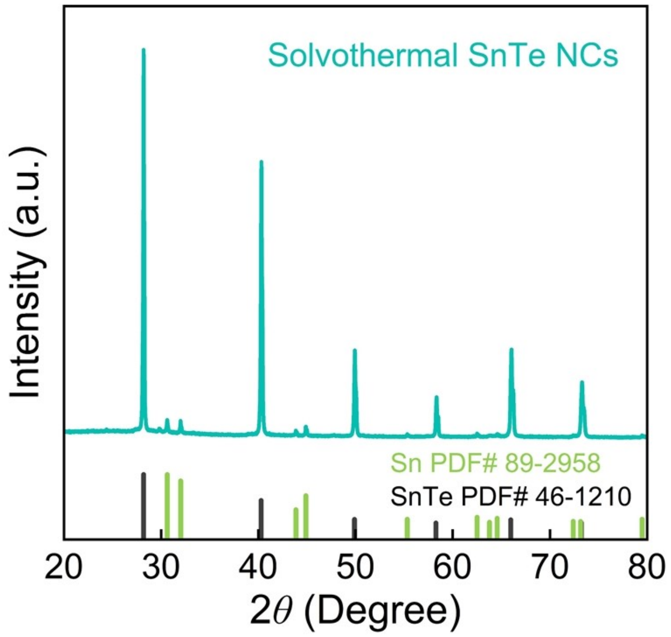

Figure S2. Room-temperature powder XRD pattern for solvothermal SnTe nanocrystals.

Discussion 1: Solvothermal synthesis of self-compensated SnTe thermoelectric nanomaterials

In this reaction, Te (1.276 g) and $SnCl_2$ (2.257 g) precursors were added to a *N,N*-Dimethylformamide (DMF)/NaOH solution, followed by the addition of sufficient sodium borohydride ($NaBH_4$, 1.893 g). According to the hard/soft acid/base (HSAB) principle[6], hard basic DMF cannot dissolve soft acidic Te. However, it can be employed as a weak complexing reagent for forming a complex with cation precursors[7] and thus controlling the dissolution rate of Sn^{2+} in a mixed solution. The presence of DMF favors the formation of finer powders. On the other hand, BH_4^- (borohydride) is a strong reducing agent - during the oxidation of one (BH_4^-), electrons are released; these electrons can reduce Te into Te^{2-}. Since the transformation process occurs in a very small layer of the Te fine powder, the SnTe obtained on it can be transferred to the solution, followed by prolonged Ostwald ripening by heating the products at 180 °C for tens of hours. Since the standard reduction potential of Sn is higher than that of $NaBH_4$ (−0.1375 V vs. −1.24 V[8]), the Sn^0 obtained via reduction with $NaBH_4$ in a homogeneous solution is thermodynamically favorable. The obtained products were collected by centrifuging and washed with deionized water and absolute ethanol at least three times, and then were vacuum dried at 60 °C for 12 h. Note that this is the first reported case of SnTe NCs enriched with Sn[9-11].

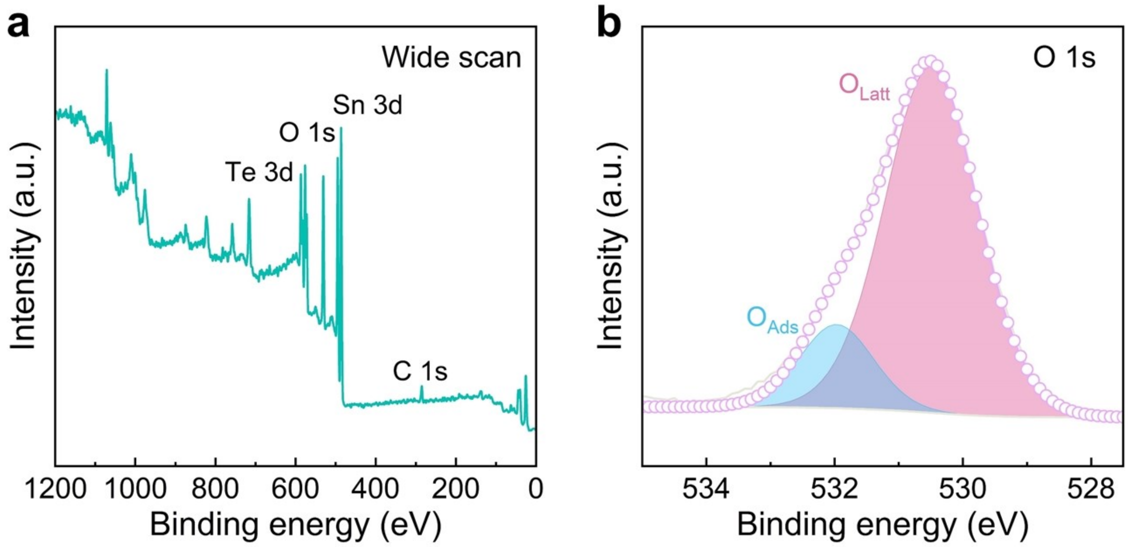

Figure S3. XPS survey spectrum for our SnTe NCs: (a) wide scan spectrum, high resolution scans of XPS spectrum for (b) O 1s.

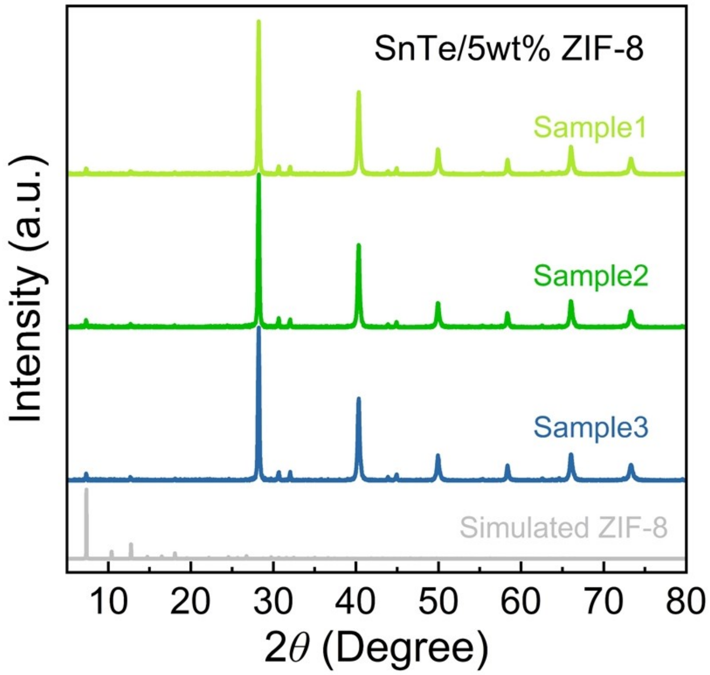

Figure S4. The repeated XRD measurements for the SnTe/5wt%ZIF-8 sample, showing good reproducibility.

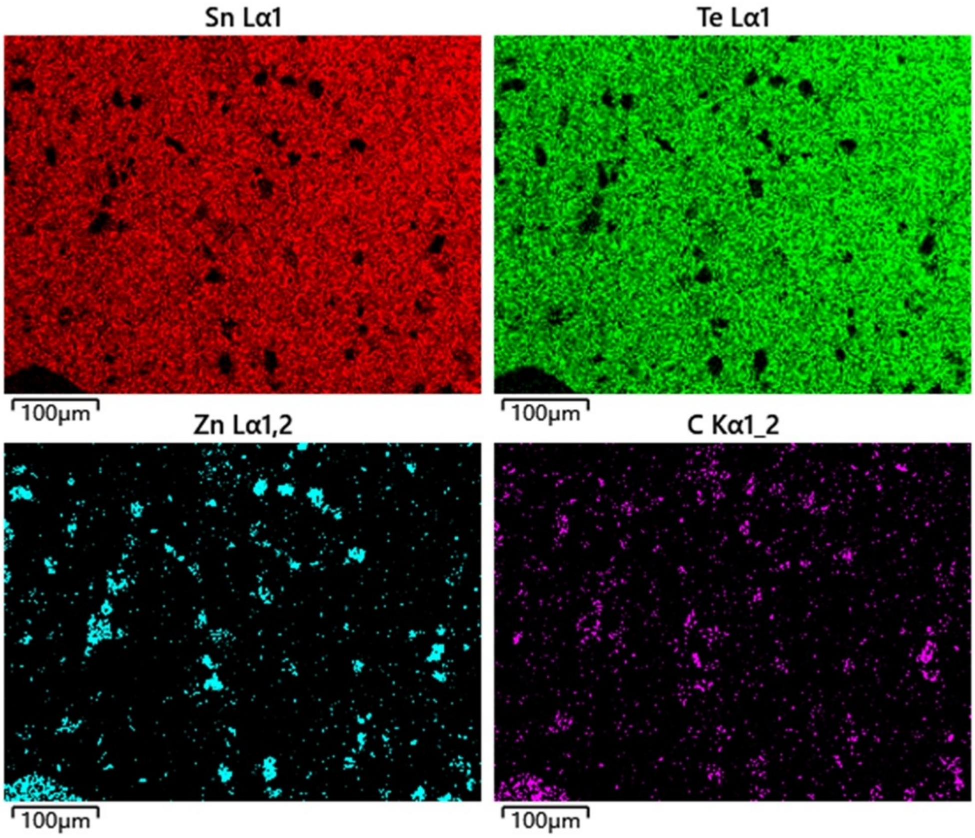

Figure S5. SEM-EDS result of the SnTe/ZIF-8 heterointerfaces, showing no discernible Zn diffusion.

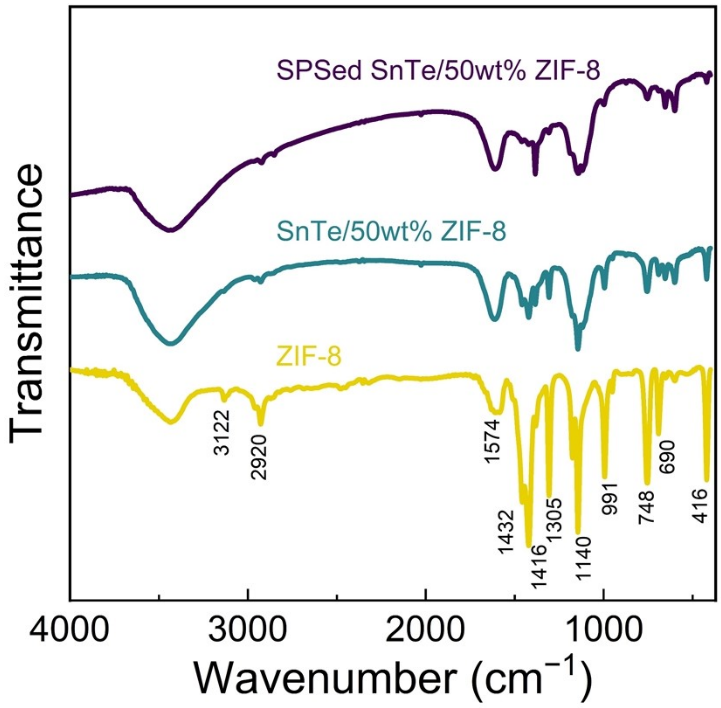

Figure S6. The FTIR spectra of ZIF-8, SnTe/50wt%ZIF-8, and as-SPSed SnTe/50wt%ZIF-8 samples.

The characteristic peaks at 2920 and 3122 cm^{-1} represent the asymmetric stretching vibrations of aliphatic C−H and aromatic rings. The absorption band at 1574 cm^{-1} is ascribed to the stretching vibration of the C=N bond, and the sharp absorption band at 416 cm^{-1} originates from the stretching vibration of Zn−N, suggesting the binding of zinc atoms to nitrogen atoms within the 2-methylimidazolate linkers. The peaks at 1305 and 1140 cm^{-1}, 1432 and 1416 cm^{-1}, and 690 cm^{-1} correspond to the bending signals, stretching vibrations, and out-of-plane bending vibrations of the imidazole rings, respectively. Analogously, the characteristic peaks at 748 and 991 cm^{-1} are connected with the bending vibrations of C−N and C−H. All the characteristic peaks of ZIF-8 are present in the FTIR spectra of SnTe/50wt%ZIF-8 and as-SPSed SnTe/50wt%ZIF-8 samples. The intensity of these peaks is diminished owing to the lower ZIF-8 content (50%).

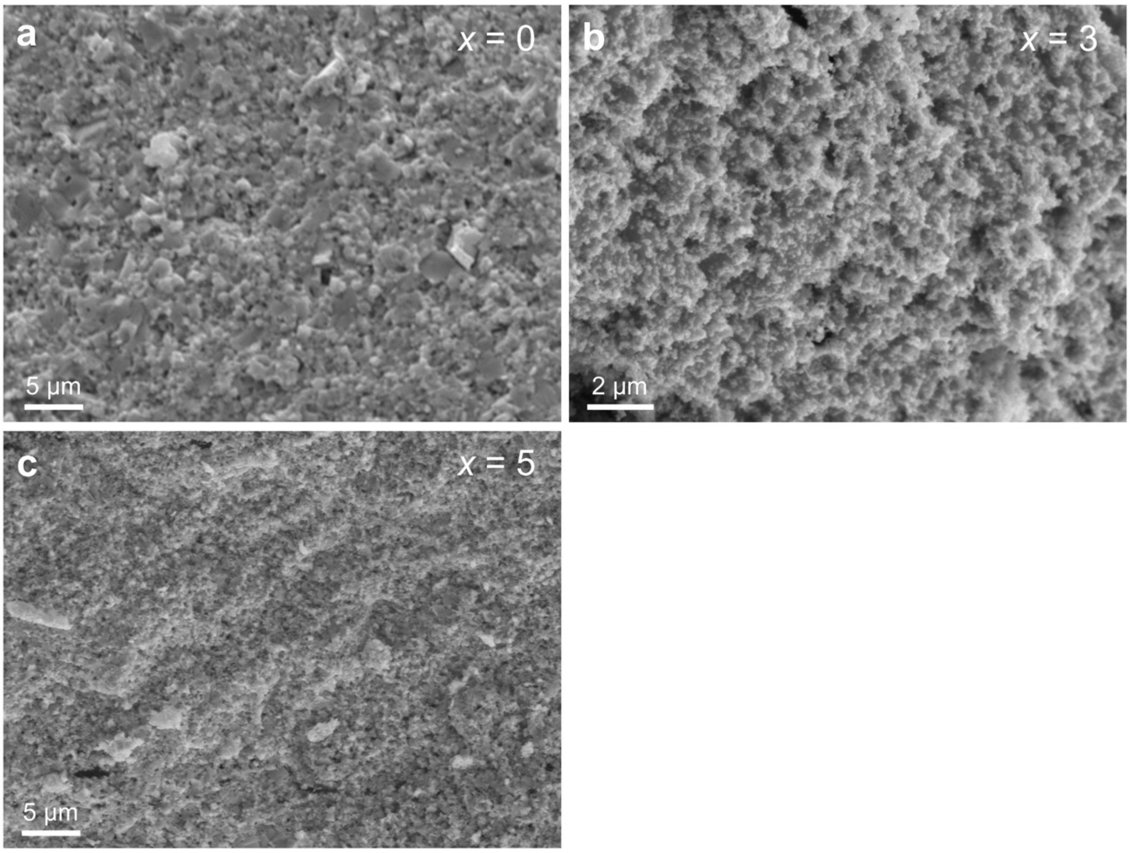

Figure S7. SEM images of the fraction surfaces for as-SPSed (a) pure SnTe, (b) SnTe/3wt%ZIF-8, and (c) SnTe/5wt%ZIF-8 samples.

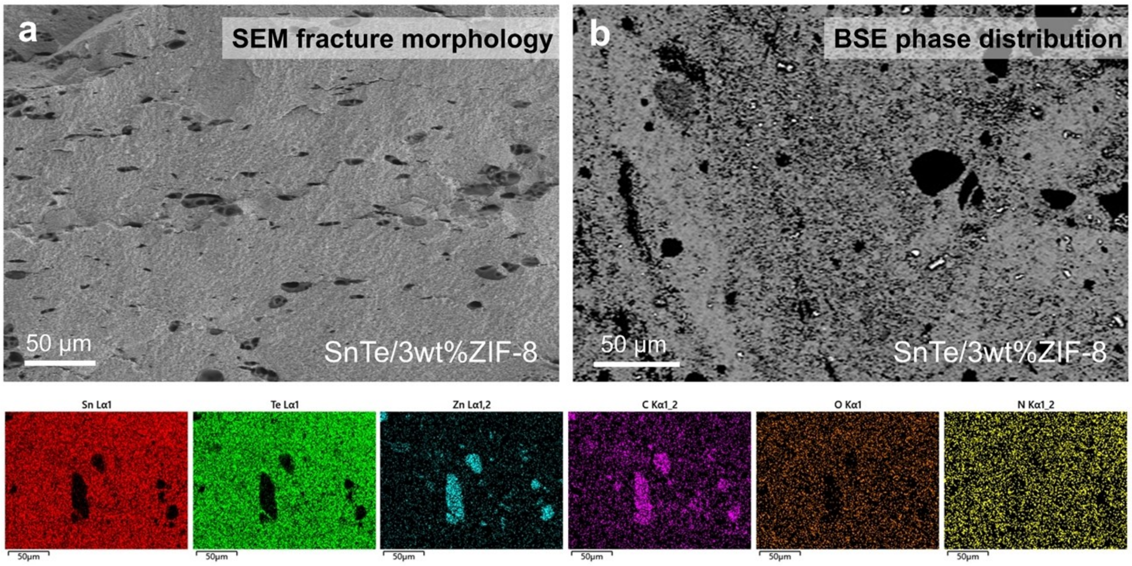

Figure S8. (a) SEM image of fractured surface for as-SPSed SnTe/3wt%ZIF-8. (b) Back-scattered electron image of polished surface for as-SPSed SnTe/3wt%ZIF-8 and EDS mappings for Sn, Te, Zn, C, N, and O elements.

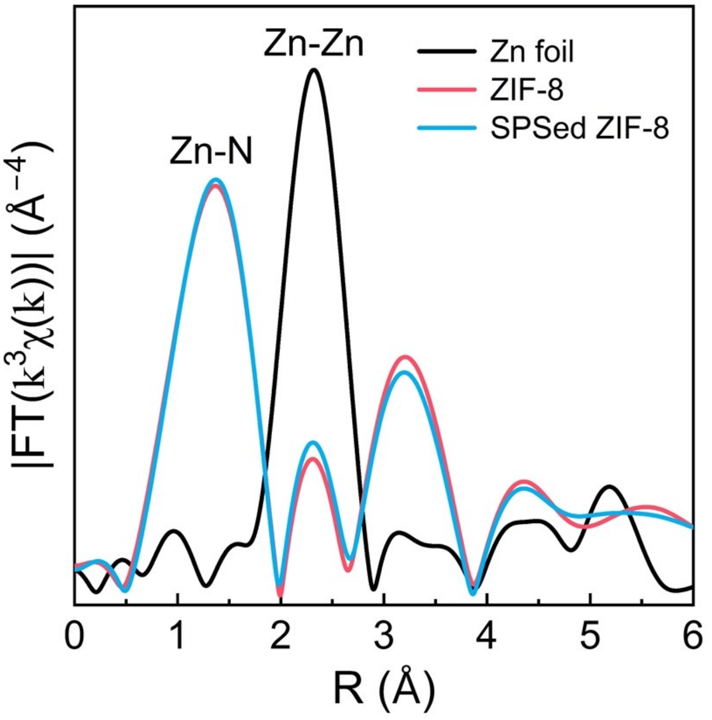

Figure S9. EXAFS Fourier-transform spectra of Zn foil, ZIF-8, and as-SPSed ZIF-8.

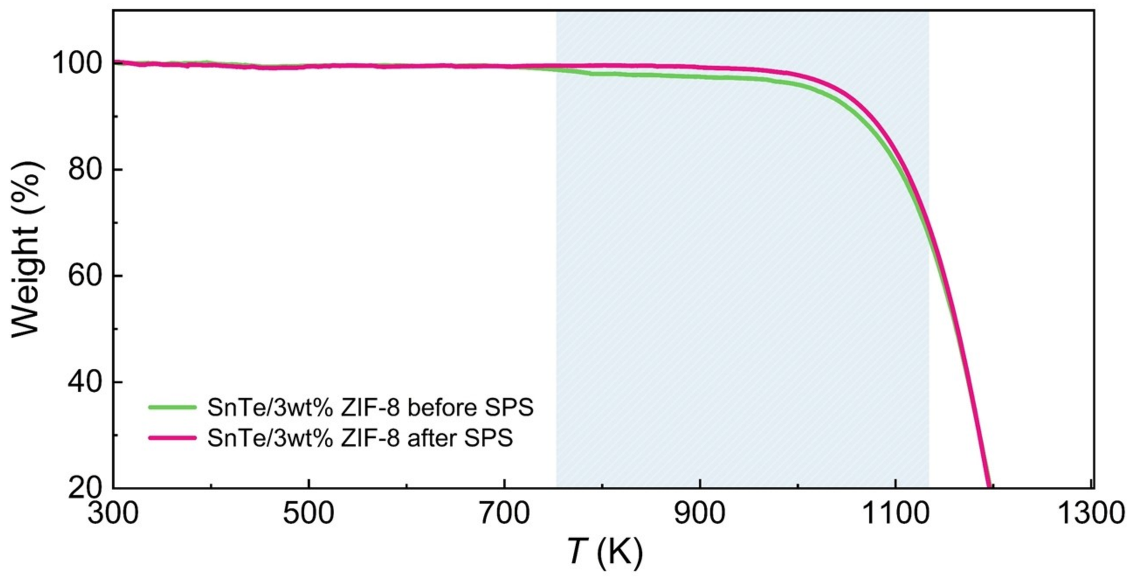

Figure S10. TG curves for the SnTe/3wt%ZIF-8 samples before and after SPS sintering.

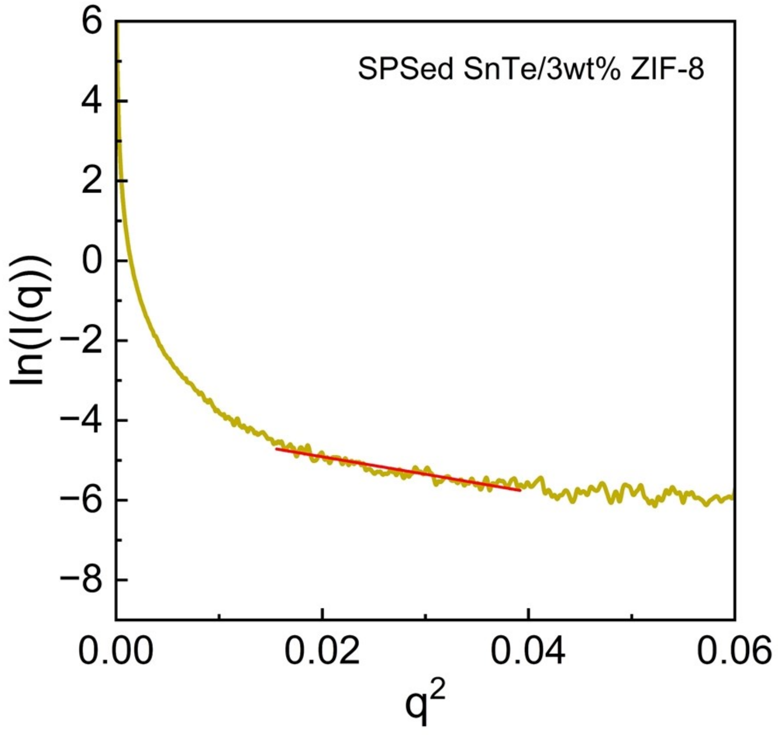

Figure S11. SAXS profile of SPSed SnTe/3wt%ZIF-8 sample. The radius of gyration (R_g) of the high-q region was determined from SAXS data[12].

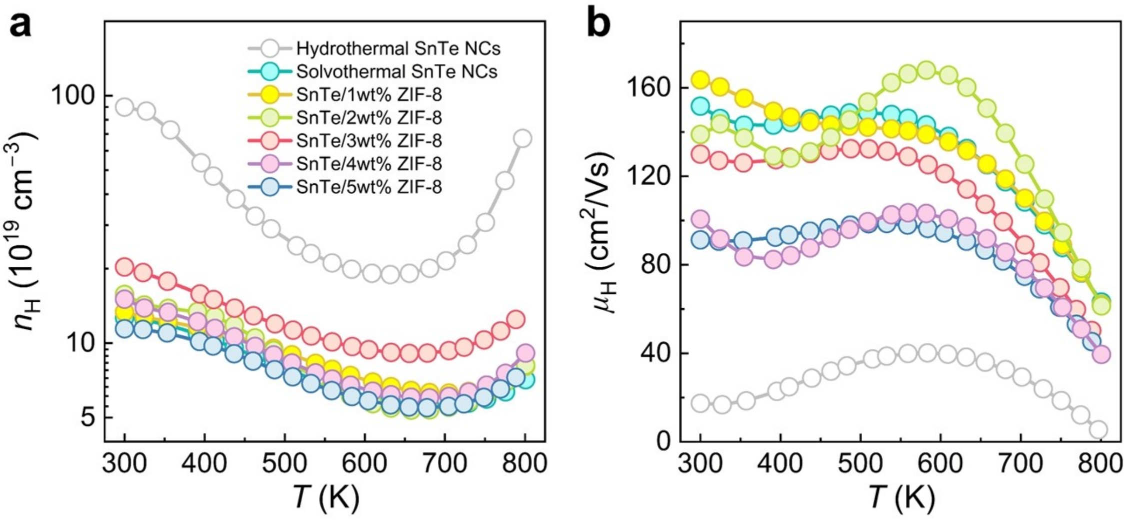

Figure S12. Temperature-dependent (a) Hall carrier concentration and (b) mobility for the SnTe/*x*wt%ZIF-8 with different compositions and the hydrothermal SnTe sample.

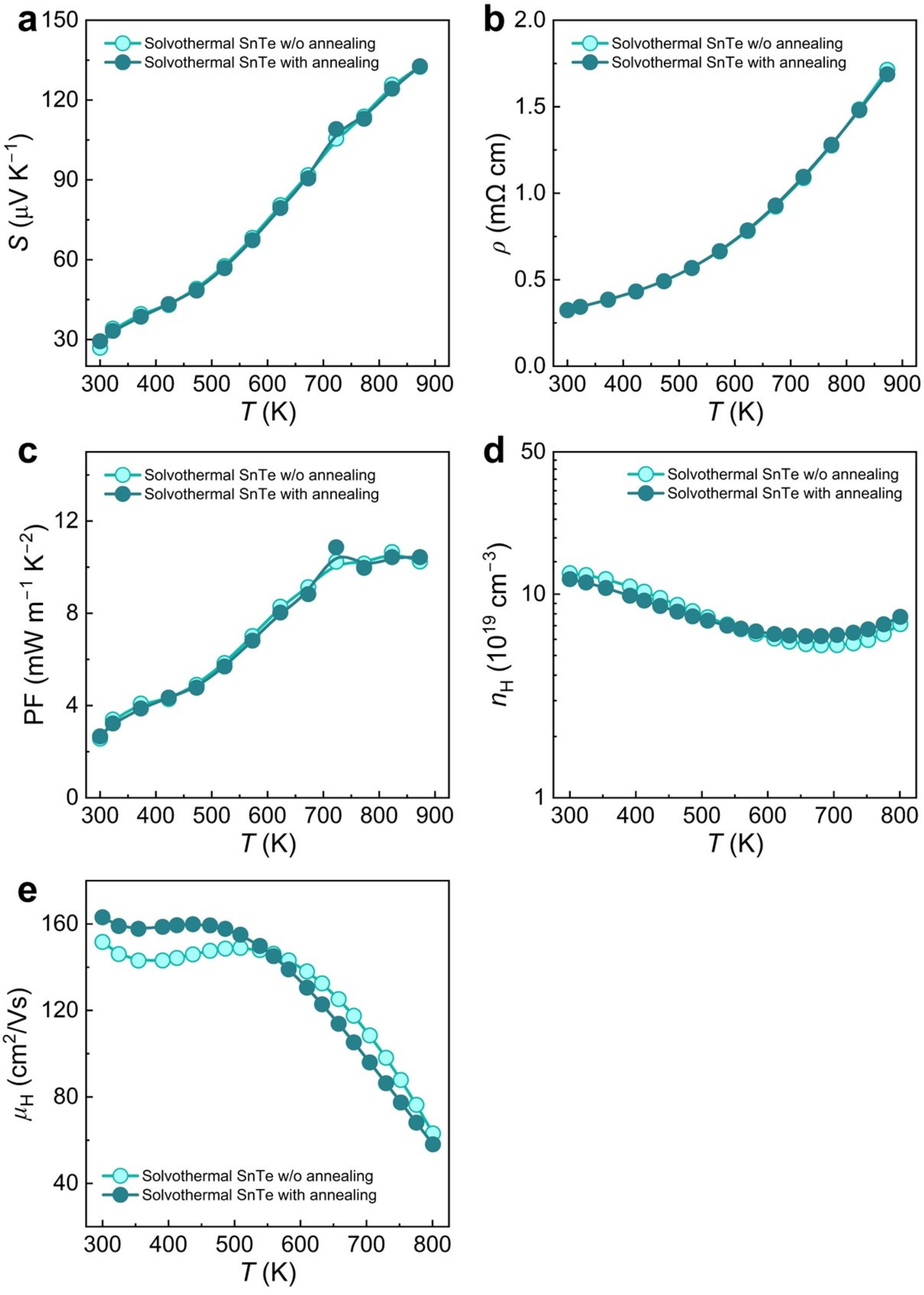

Figure S13. The electrical properties for solvothermal prepared SnTe with and without annealing at 873 K for 3 h, showing a highly stable performance. Temperature dependence of (a) Seebeck coefficient, (b) electrical resistivity, (c) PF, (d) Hall carrier concentration, and (e) Hall mobility. Compared to solvothermal SnTe before annealing, the transport properties of the annealed SnTe remain largely unchanged.

Discussion 2: Snyder's two-phase model for energy filtering description in a heterogeneous material with GBs

The Seebeck coefficient (*S*) is determined via the average entropy transported per charge carrier. Thus, *S* will increase as charge-carriers with greater entropy contribute more to the total electrical conductivity[13]. Introduction of energetic barriers to restrict the transport of lower-energy charge-carriers, while allowing higher-energy carriers to pass unimpeded, is one means by which the average entropy transported per charge-carrier can be increased, referred to as energy filtering[14]. Thus, the high energy carriers contribute more to the Seebeck coefficient than the low energy carriers. In a polycrystalline material with grain boundaries (GBs), carrier transport behaviors are different in grains and at grain boundaries. A potential barrier at a grain boundary would induce both the GB band offset (ΔE) and the interfacial resistance. Specifically, the band offset (ΔE) between the valence band maximum (VBM) of the grain and the grain boundary (GB) can act as the hole carrier filter (Figure S14). By changing the band structure at the grain boundaries, the low energy carriers can be preferentially "filtered out", therefore increasing the magnitude of the Seebeck coefficient (*S*). The potential barrier, or band offset at the GB should lead to the grain boundary region having a larger magnitude of the Seebeck coefficient (*S*) compared to the rest of the bulk (i.e., $S_{GB}>S_{grain}$).

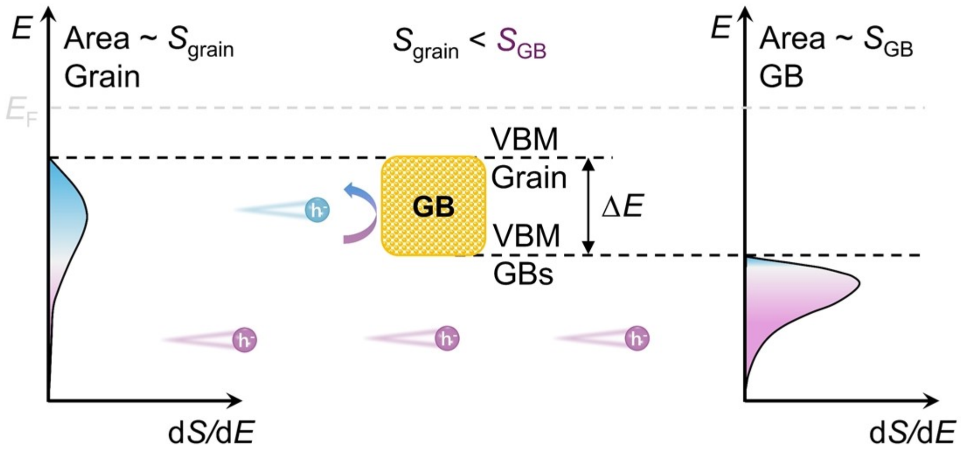

Figure S14. Schematic diagram of the energy filtering effect.

As a result, the introduction of high-density resistive interfaces can boost the contribution of the interfacial Seebeck coefficient to the total Seebeck coefficient[13]. For

a heterogeneous material of grain and GB phase with a total temperature drop ΔT_{total}, the obvious overall S_{total} will be:

$$S_{total} = S_{grain} + (S_{GB} - S_{grain})\frac{\Delta T_{GB}}{\Delta T_{total}} \tag{S1}$$

Here, S_{grain} and S_{GB} are the Seebeck coefficients of the grain and GB phase, and ΔT_{GB} is the temperature drop at the GBs. For $|S_{GB}| > |S_{grain}|$, a larger fractional temperature drop across the GB regions $\Delta T_{GB}/\Delta T_{total}$ will lead to a larger magnitude of the Seebeck coefficient ($|S_{total}|$). Importantly, $\Delta T_{GB}/\Delta T_{total}$ can be enhanced by increasing the interfacial Kapitza resistance ($\rho_{Kapitza}$)[15]. Kapitza resistance ($\rho_{Kapitza}$) is a measure of the resistance of an interface to the transport of heat through it. The Kapitza resistance ($\rho_{Kapitza}$) is commonly observed in composite material systems due to differences in the physical properties of constituent materials and increases with the high dissimilarity in sound velocity and phonon density of states[16].

$$\frac{\Delta T_{GB}}{\Delta T_{total}} = \frac{1}{\dfrac{d}{\kappa_{grain}\rho_{Kapitza}} + 1} \tag{S2}$$

Here, κ_{grain} is the thermal conductivity of the grain phase. Therefore, our SnTe/$x$wt%ZIF-8 nanocomposites are conducive to realizing enhanced total Seebeck coefficient (S_{total}) via the dual-barrier-induced band offset and Kapitza resistance.

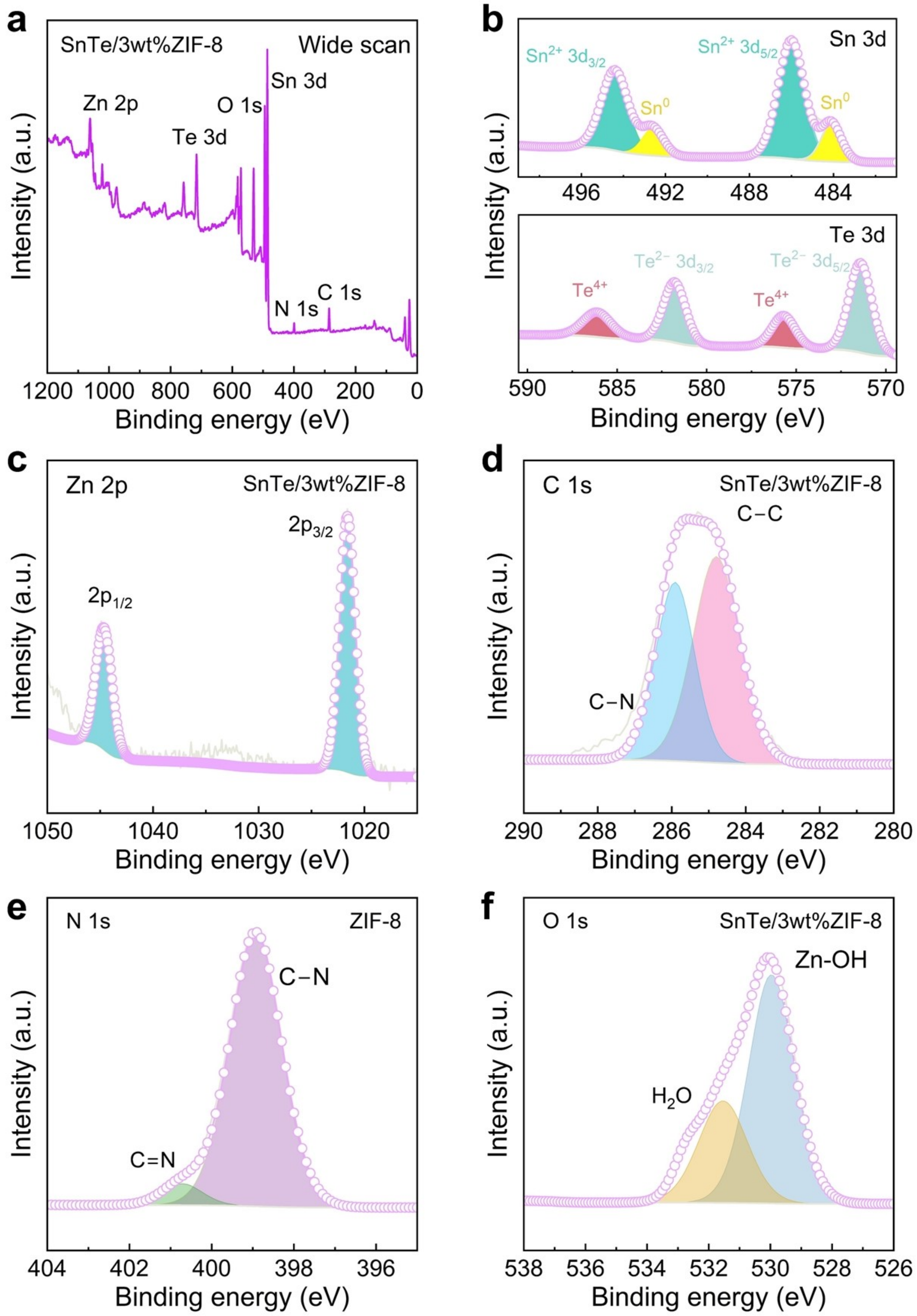

Figure S15. XPS spectra of wide scan (a) for as-SPSed SnTe/3wt%ZIF-8 sample and high-resolution scan for (b) Sn 3d and Te 3d, (c) Zn 2p, (d) C 1s, (e) N 1s, and (f) O 1s.

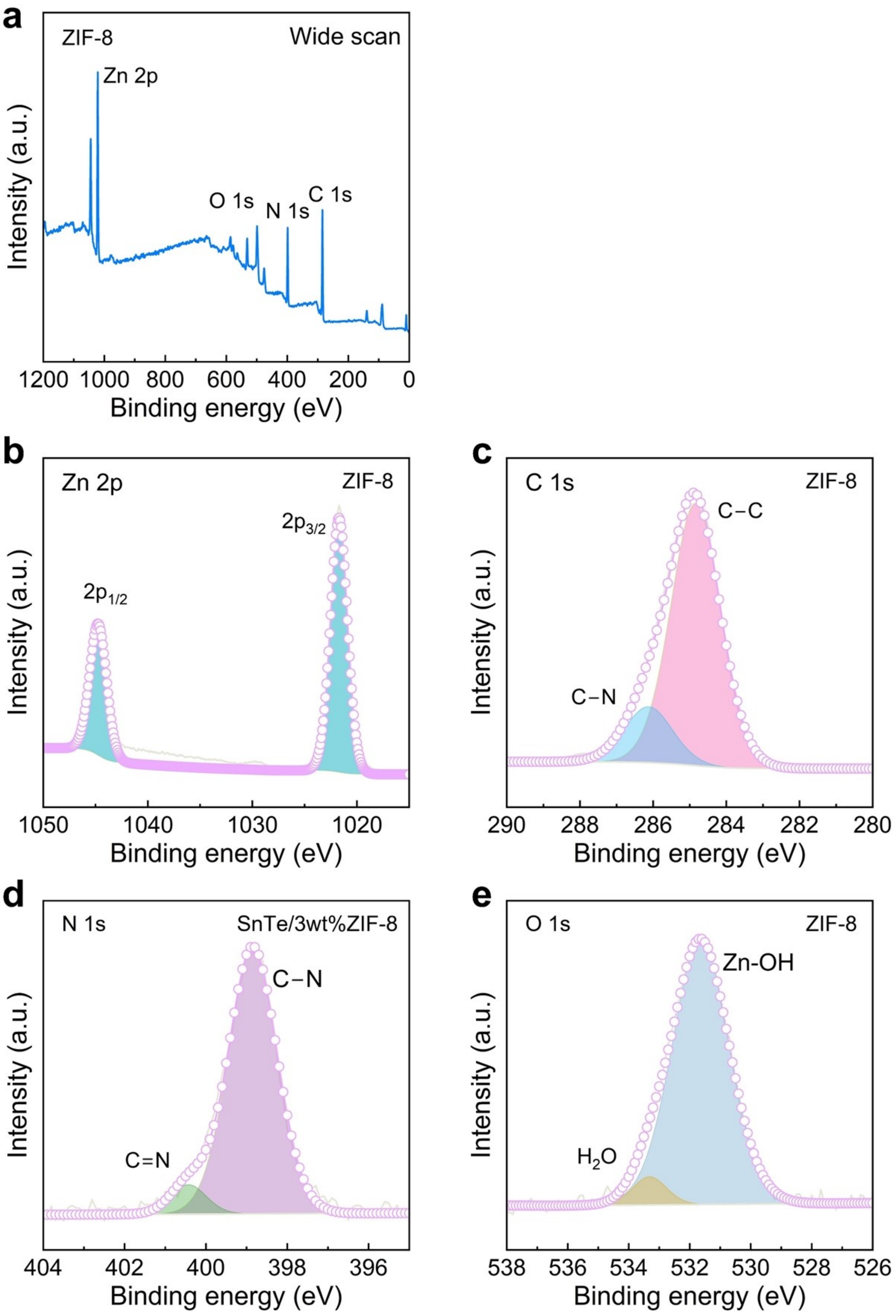

Figure S16. XPS spectra of wide scan (a) for as-SPSed ZIF-8 sample and high-resolution scan for (b) Zn 2p, (c) C 1s, (d) N 1s, and (e) O 1s.

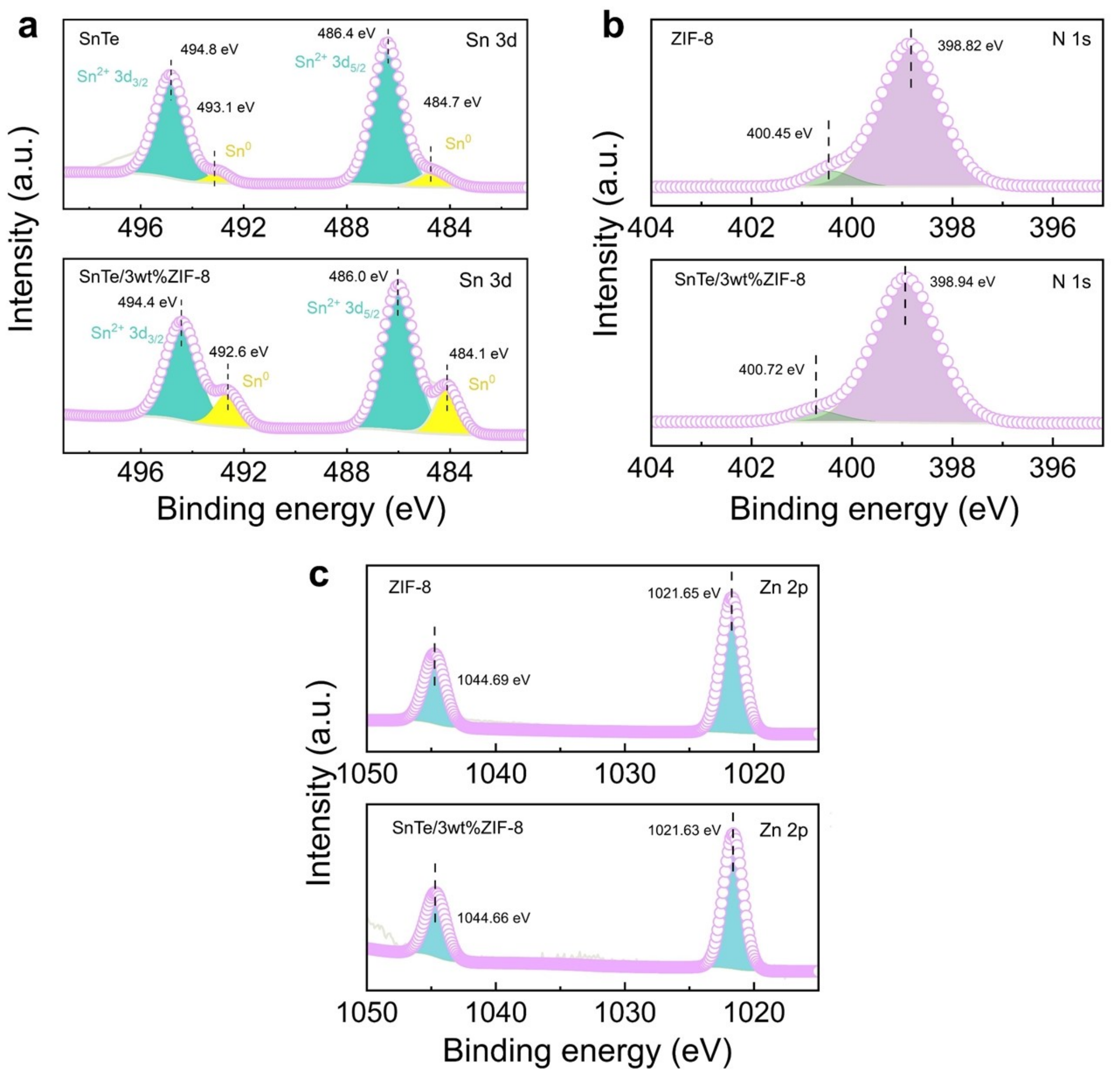

Figure S17. XPS high-resolution scan for (a) Sn 3d, (b) N 1s, and (d) Zn 2p for as-SPSed SnTe, as-SPSed ZIF-8 and as-SPSed SnTe/3wt%ZIF-8 samples.

The XPS technique was carried out to further reveal the chemical composition and electronic structure of SnTe/ZIF-8 heterostructure. We compared the XPS spectra of as-SPSed SnTe, as-SPSed ZIF-8, and as-SPSed SnTe/3wt%ZIF-8 samples (Figure S15−S17). The XPS spectra illustrate that the SnTe/3wt%ZIF-8 sample contains elements such as Sn, Te, Zn, C, N and O (Figure S15). The peaks at 494.8 eV and 486.4 eV correspond to Sn $3d_{3/2}$ and $3d_{5/2}$ of SnTe, while in the SnTe/3wt%ZIF-8 sample, the Sn $3d_{3/2}$ and $3d_{5/2}$ peaks appear at 494.4 eV and 486.0 eV, respectively (Figure S17a). Compared with pure SnTe, after constructing the heterostructure with ZIF-8, the Sn 3d peaks shift toward lower binding energy by 0.4 eV. In the SnTe/3wt%ZIF-8 nanocomposite, compared with ZIF-8, N 1s peaks tend to shift toward higher binding energy (Figure S17b). The Zn 2p peaks of SnTe/3wt%ZIF-8 are located at 1021.62 eV, showing a negligible shift compared to the pure ZIF-8 sample (Figure S17c). These XPS

results confirmed the formation of Sn−N bonding, indicating electronic interaction at the SnTe-ZIF-8 interface, together suggesting evident interfacial charge redistribution after heterostructure construction. Such electronic rearrangement is conducive to the establishment of a carrier energy-filtering effect.

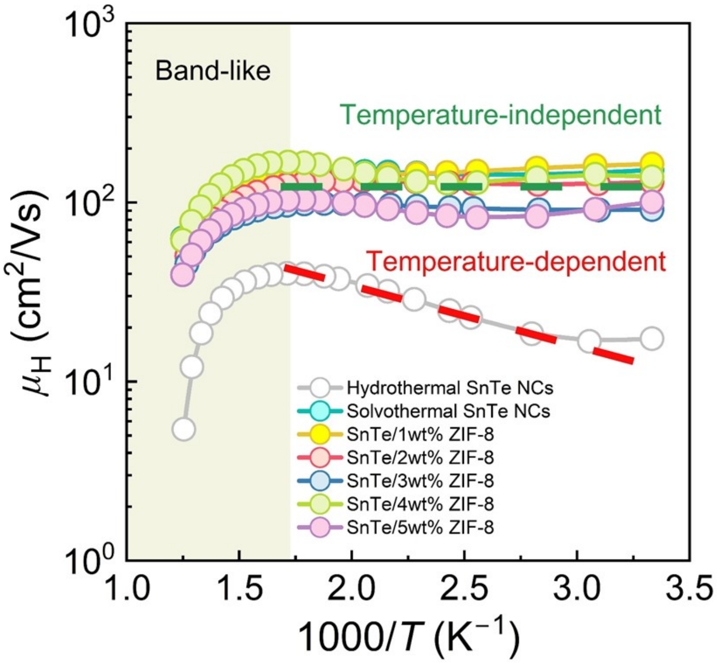

Figure S18. Arrhenius plots of the temperature dependence of the mobility of SnTe/$x$wt%ZIF-8 (x = 0, 1, 2, 3, 4, and 5) and hydrothermal SnTe.

For temperature < 580 K, the mobilities of SnTe/$x$wt%ZIF-8 are constant even with varying temperature. By contrast, the mobility of hydrothermal SnTe (without organics/ZIF-8) decreases upon lowering the temperature from 580 to 327 K. The most plausible explanation for the observed temperature-independent mobility is tunneling transport[17], which may occur at the SnTe-organic interface. Moreover, the increase in mobility with decreasing temperature observed in region yellow of Figure S18 is a "bandlike" transport[18] for all SnTe samples.

Discussion 3: Calculation of lattice thermal conductivity (κ_L)

The material studied here is inherently complicated, and a simple, systematic thermal model is required to separate the different contributions to the reduction in κ_L. To describe the effects of having a mixture of two materials (in this case, the experimental material and air), we use effective medium theory (EMT) to analyze the thermal conductivity of porous SnTe/ZIF-8 materials. In the EMT model, an assumption is that for a heterogeneous material, in which the two components should be distributed randomly, with neither phase being necessarily continuous or dispersed. Plus, either component may form continuous heat conduction pathways, depending on the relative amounts of the components. The absence of thermal conduction within the pores is described classically by the EMT described above, leading to a reduction in lattice thermal conductivity described by $\kappa_{L,\,R} = f_\kappa(\varepsilon)\kappa_{L,\,dense} = (1-3\varepsilon/2)\kappa_{L,\,dense}$, where $\kappa_{L,\,dense}$ is the κ_L of the imaginary fully dense material, ε is porosity volume fraction.

To further elucidate the influence of various phonon scattering mechanisms on $\kappa_{L,dense}$-reduction for fully dense SnTe/3wt%ZIF-8, we evaluated the temperature-dependent $\kappa_{L,dense}$ according to the modified Debye−Callaway model from the following equation:[19, 20]

$$\kappa_{L,dense} = \frac{k_B}{2\pi^2 v_s}\left(\frac{k_B T}{\hbar}\right)^3 \int_0^{\Theta_D/T} \frac{x^4 e^x}{\tau_{tot}(x)^{-1}\left(e^x-1\right)^2} dx \quad (S3)$$

$$\text{with } x = \frac{\hbar\omega}{k_B T}$$

Since the integral of spectral lattice thermal conductivity ($\kappa_{s,dense}$) with respect to the phonon frequency (ω) is equivalent to the corresponding $\kappa_{L,dense}$, the $\kappa_{s,dense}$ can be calculated according to equation (S4):

$$\kappa_{s,dense} = \frac{k_B}{2\pi^2 v_s}\left(\frac{k_B T}{\hbar}\right)^3 \frac{\tau_{tot}(x) x^4 e^x}{\left(e^x-1\right)^2} \quad (S4)$$

Here, k_B, $\hbar$, v_s, Θ_D, τ_{tot}, x, and ω are the Boltzmann constant, reduced Planck's constant, average sound velocity, Debye temperature, total phonon relaxation time, reduced frequency, and phonon frequency, respectively. In this work, we take into account four scattering mechanisms for the calculation of κ_L, including Umklapp processes (U),

heterostructure interface (I), grain boundaries (B), and sub-nano pores (SP). The corresponding total relaxation time τ_{tot} can be calculated based on the Matthiessen's rule:

$$\tau_{tot}^{-1} = \sum_i \tau_i^{-1} = \tau_U^{-1} + \tau_I^{-1} + \tau_B^{-1} + \tau_{SP}^{-1} \quad \text{(S5)}$$

The phonon relaxation time of Umklapp processes scattering can be obtained from:

$$\tau_U^{-1} = \frac{\hbar \gamma^2 T}{\bar{M} v_s^2 \Theta_D} \omega^2 e^{-\Theta_D/3T} \quad \text{(S6)}$$

The relaxation time associated with grain boundaries scattering is expressed as:

$$\tau_B^{-1} = \frac{v_s}{d_{avg}} \quad \text{(S7)}$$

When regarding the ZIF-8 and sub-nano pores as a solid spherical second phase, their relaxation times can be written as:

$$\tau^{-1} = v_s N_p \left[\left(2\pi R^2\right)^{-1} + \left(\frac{4}{9}\pi R^2 \left(\frac{\Delta D}{D}\right)^2 \left(\frac{\omega R}{v_s}\right)^4 \right)^{-1} \right]^{-1} \quad \text{(S8)}$$

In the above equations, γ is the Grüneisen parameter, $\bar{M}$ is the average atomic mass, d_{avg} is the average grain size of the samples, v_s is the average sound velocity, Θ_D is the Debye temperature, N_p is the number density of the second phase, R is the average radius for the second phase, D is the SnTe matrix density, ΔD is the density difference between matrix and second phase (the mass density of pores is considered as zero).

The aforementioned parameters used for the Debye−Callaway modeling are listed in Table S1 below.

Table S1. The detailed parameters used to model $\kappa_{L,dense}$ of SnTe/3wt%ZIF-8 sample.

Parameter	symbol	value (unit)
Grüneisen parameter	γ	1.44
Debye temperature	Θ_D	148 K
Average sound velocity	v_s	2175 m/s
Average atomic mass	$\bar{M}$	1.97×10^{-25} kg

Matrix density	D_{SnTe}	6.13 g cm^{-3}
ZIF-8 density	$D_{ZIF\text{-}8}$	0.95 g cm^{-3}
Average SnTe grain size	d_{avg}	450 nm
Average radius for pores	R_{pore}	0.475 nm
Average radius for ZIF-8 phase	$R_{ZIF\text{-}8}$	190 nm

Table S2. The experimental and calculated results of lattice thermal conductivity κ_L based on the effective medium theory (EMT) model for SnTe/3wt%ZIF-8 sample (the void fraction is set as 8%).

Temperature (K)	Experimental value (W m^{-1} K^{-1})	Calculated value (W m^{-1} K^{-1})
300	1.07	1.44
873	0.18	0.31

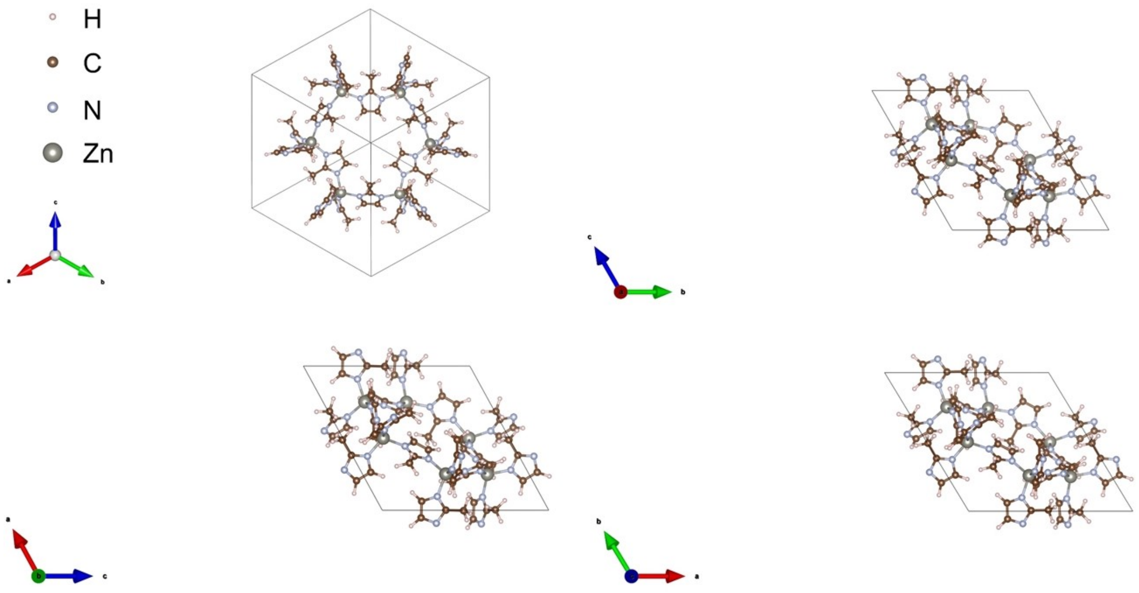

Figure S19. The DFT-relaxed structure of ZIF-8.

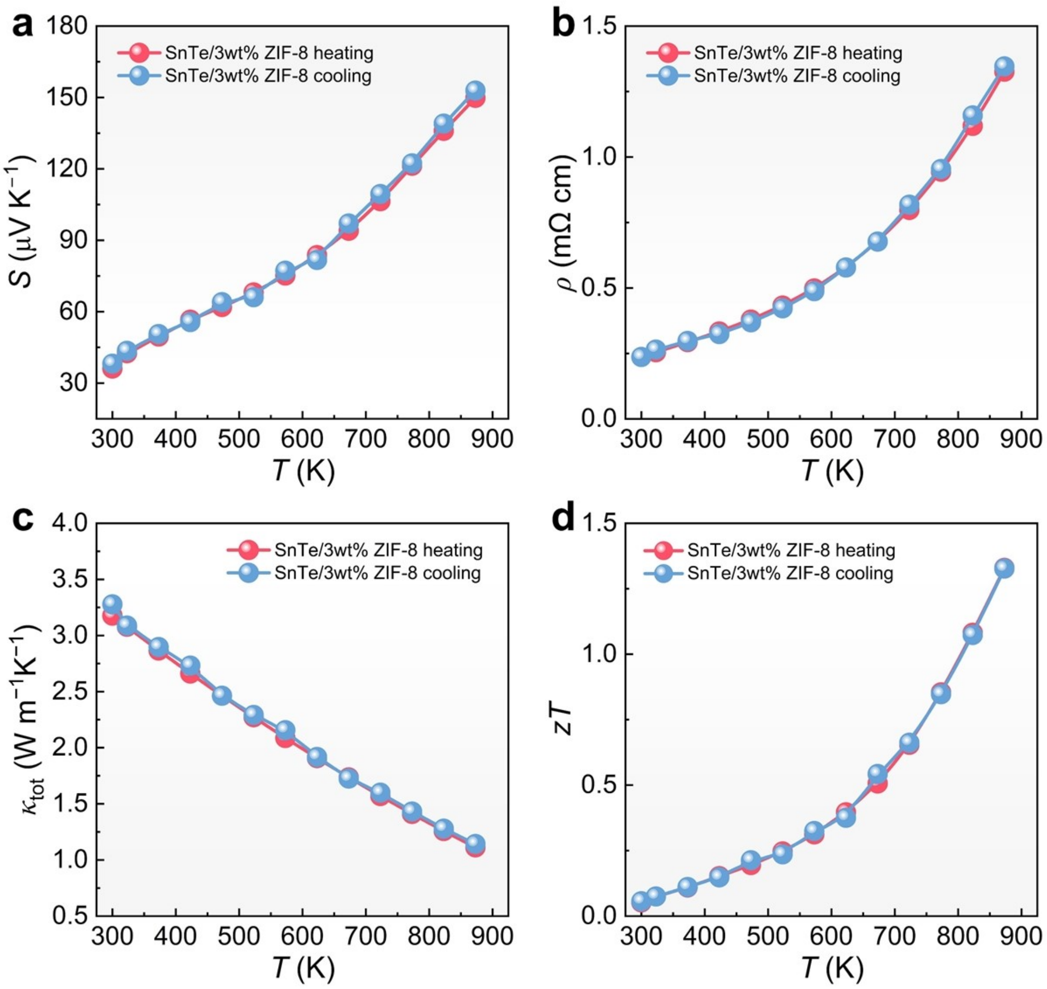

Figure S20. The repeated measurements of thermoelectric properties for SnTe/3wt%ZIF-8, showing a highly reproducible and stable performance. Temperature dependence of (a) Seebeck coefficient, (b) electrical resistivity, (c) total thermal conductivity, and (d) *zT* values.

References

1. W. Jia, Z. Cao, L. Wang, J. Fu, X. Chi, W. Gao and L.-W. Wang, *Computer Physics Communications*, 2013, **184**, 9-18.
2. W. Jia, J. Fu, Z. Cao, L. Wang, X. Chi, W. Gao and L.-W. Wang, *Journal of Computational Physics*, 2013, **251**, 102-115.
3. A. Togo and I. Tanaka, *Scripta Materialia*, 2015, **108**, 1-5.
4. J. P. Perdew, K. Burke and M. Ernzerhof, *Physical Review Letters*, 1996, **77**, 3865-3868.
5. H. J. Monkhorst and J. D. Pack, *Physical Review B*, 1976, **13**, 5188-5192.
6. R. G. Pearson, *Journal of the American Chemical Society*, 1963, **85**, 3533-3539.
7. C. Niu, Y. Gong, R. Qiu, Q. Zhu, Y. Zhou, S. Hao, W. Yan, W. Huang and H. Xin, *Journal of Materials Chemistry A*, 2021, **9**, 12981-12987.
8. A. Trifonova, M. Wachtler, M. Winter and J. O. Besenhard, *Ionics*, 2002, **8**, 321-328.
9. B.-Z. Tian, J. Chen, X.-P. Jiang, J. Tang, D.-L. Zhou, Q. Sun, L. Yang and Z.-G. Chen, *ACS Applied Materials & Interfaces*, 2021, **13**, 50057-50064.
10. R. Moshwan, W.-D. Liu, X.-L. Shi, Y.-P. Wang, J. Zou and Z.-G. Chen, *Nano Energy*, 2019, **65**, 104056.
11. L. Wang, S. Chang, S. Zheng, T. Fang, W. Cui, P.-p. Bai, L. Yue and Z.-G. Chen, *ACS Applied Materials & Interfaces*, 2017, **9**, 22612-22619.
12. S. Lombardo, H. Khalili, S. Yu, S. Mukherjee, K. Nygård, Z. Bacsik and A. P. Mathew, *ACS Applied Materials & Interfaces*, 2025, **17**, 48976-48988.
13. Y. Lin, M. Wood, K. Imasato, J. J. Kuo, D. Lam, A. N. Mortazavi, T. J. Slade, S. A. Hodge, K. Xi, M. G. Kanatzidis, D. R. Clarke, M. C. Hersam and G. J. Snyder, *Energy & Environmental Science*, 2020, **13**, 4114-4121.
14. Z. Liang, M. J. Boland, K. Butrouna, D. R. Strachan and K. R. Graham, *Journal of Materials Chemistry A*, 2017, **5**, 15891-15900.
15. G. L. Pollack, *Reviews of Modern Physics*, 1969, **41**, 48-81.
16. P. K. Schelling, S. R. Phillpot and P. Keblinski, *Journal of Applied Physics*, 2004, **95**, 6082-6091.
17. M. Grifoni and P. Hänggi, *Physics Reports*, 1998, **304**, 229-354.
18. H. Geng, Q. Peng, L. Wang, H. Li, Y. Liao, Z. Ma and Z. Shuai, *Advanced Materials*, 2012, **24**, 3568-3572.
19. J. Callaway, *Physical Review*, 1959, **113**, 1046-1051.
20. H.-S. Kim, S. I. Kim, K. H. Lee, S. W. Kim and G. J. Snyder, *physica status solidi (b)*, 2017, **254**, 1600103.
